# Intravenous Delayed Gadolinium-Enhanced MR Imaging of the Endolymphatic Space: A Methodological Comparative Study

**DOI:** 10.3389/fneur.2021.647296

**Published:** 2021-04-22

**Authors:** Rainer Boegle, Johannes Gerb, Emilie Kierig, Sandra Becker-Bense, Birgit Ertl-Wagner, Marianne Dieterich, Valerie Kirsch

**Affiliations:** ^1^Department of Neurology, University Hospital, Ludwig-Maximilians-Universität, Munich, Germany; ^2^German Center for Vertigo and Balance Disorders-IFB (Integriertes Forschungs- und Behandlungszentrum), University Hospital, Ludwig-Maximilians-Universität, Munich, Germany; ^3^Graduate School of Systemic Neuroscience (GSN), Ludwig-Maximilians-Universität, Munich, Germany; ^4^Department of Radiology, The Hospital for Sick Children, University of Toronto, Toronto, ON, Canada; ^5^Department of Radiology, University Hospital, Ludwig-Maximilians-Universität, Munich, Germany; ^6^Munich Cluster for Systems Neurology (SyNergy), Munich, Germany

**Keywords:** endolymphatic hydrops, endolymphatic space, inner ear imaging, gadolinium based contrast agent, intravenous, convolutional neural network, deep learning, volumetric local thresholding

## Abstract

*In-vivo* non-invasive verification of endolymphatic hydrops (ELH) by means of intravenous delayed gadolinium (Gd) enhanced magnetic resonance imaging of the inner ear (iMRI) is rapidly developing into a standard clinical tool to investigate peripheral vestibulo-cochlear syndromes. In this context, methodological comparative studies providing standardization and comparability between labs seem even more important, but so far very few are available. One hundred eight participants [75 patients with Meniere's disease (MD; 55.2 ± 14.9 years) and 33 vestibular healthy controls (HC; 46.4 ± 15.6 years)] were examined. The aim was to understand (i) how variations in acquisition protocols influence endolymphatic space (ELS) MR-signals; (ii) how ELS quantification methods correlate to each other or clinical data; and finally, (iii) how ELS extent influences MR-signals. Diagnostics included neuro-otological assessment, video-oculography during caloric stimulation, head-impulse test, audiometry, and iMRI. Data analysis provided semi-quantitative (SQ) visual grading and automatic algorithmic quantitative segmentation of ELS area [2D, mm^2^] and volume [3D, mm^3^] using deep learning-based segmentation and volumetric local thresholding. Within the range of 0.1–0.2 mmol/kg Gd dosage and a 4 h ± 30 min time delay, SQ grading and 2D- or 3D-quantifications were independent of signal intensity (SI) and signal-to-noise ratio (SNR; FWE corrected, *p* < 0.05). The ELS quantification methods used were highly reproducible across raters or thresholds and correlated strongly (0.3–0.8). However, 3D-quantifications showed the least variability. Asymmetry indices and normalized ELH proved the most useful for predicting quantitative clinical data. ELH size influenced SI (cochlear basal turn *p* < 0.001), but not SNR. SI could not predict the presence of ELH. In conclusion, (1) Gd dosage of 0.1–0.2 mmol/kg after 4 h ± 30 min time delay suffices for ELS quantification. (2) A consensus is needed on a clinical SQ grading classification including a standardized level of evaluation reconstructed to anatomical fixpoints. (3) 3D-quantification methods of the ELS are best suited for correlations with clinical variables and should include both ears and ELS values reported relative or normalized to size. (4) The presence of ELH increases signal intensity in the basal cochlear turn weakly, but cannot predict the presence of ELH.

## Introduction

*In-vivo* non-invasive verification of endolymphatic hydrops (ELH) by means of delayed gadolinium (Gd) enhanced magnetic resonance imaging of the inner ear (iMRI) is rapidly developing into a standard clinical tool to investigate episodic vertigo (1–3). This is due to iMRI allowing pre-mortem detection of ELH for the first time (4, 5), demonstrating that ELH is not pathognomonic to Menière's disease (MD) (6–8), but rather a concomitant that can be found in various etiologies of episodic vertigo (9–13). Consequently, the clinical prevalence and pathophysiological significance of ELH has yet to be conclusively clarified. Understanding the underpinnings of the ELH syndrome requires a systematic investigation of pathologies involving endolymphatic space (ELS) changes as well as its base physiological condition.

Data acquisition protocols have undergone a continuous optimization of MR sequences (14, 15), as well as a steady minimization of procedural invasiveness (via a shift from intratympanic to intravenous application), duration and Gd dosage (16–18). A variety of cochlear and vestibular ELH quantification conventions have been suggested, including ELS semi-quantitative visual grading (19–25), manual measurement (26–28), semi-automatic (29, 30), and automatic algorithmic area ratio (AR), and volumetric segmentation (31, 32).

Given the plurality of approaches (some manual, some algorithmic), not all published results are inherently comparable. ELH features may vary greatly depending on ELS classification (for an overview, see Table 1) and data analysis choices. In this context, methodological comparative studies providing normalization and standard values between the methods and classifications used seem all the more important but still remain rare. On this note, this study aims to investigate the following questions:

*(i)* How variations in data acquisition protocols, such as Gd dosage or time delay, influence signal intensity (SI) and signal-to-noise ratio (SNR) within the ELS.

*(ii*) How ELH measures correlate with each other, as well as with clinical symptoms or neurophysiological testing.

*(iii)* How ELH influences SNR and SI within the ELS.

**Table 1:** Semi-quantitative (SQ) grading conventions at a glance.

	**Nakashima et al. (19)**	**Gürkov et al. (24) Yang et al. (25)**	**Baráth et al. (20)**	**Attyé et al. (81)**	**Kirsch et al. (22) Boegle et al. (present data)**	**Bernaerts et al. (21) Bernaerts and de Foer (23)**
**COCHLEA**						
*Slice of evaluation*	*Midmodiolar level*	*Not specified*	*Midmodiolar level*	*Same as Nakashima et al. (19)*	*Midmodiolar level*	*Midmodiolar level*
Grade 0	No displacement of RM, interscalar septum, scala tympani, cochlear duct, scala vestibuli visible	No enlargement of ELS, PLS is clearly visible	No displacement of RM, interscalar septum, scala tympani, cochlear duct, scala vestibuli visible		**“X-mas tree”** made of circles with **“very thin, clear, hypointense line”** (cp. Figure 1A).	**“Very thin, clear, hypointense line”** (=non-enhancing scala media or ELS) between clearly enhancing scala vestibuli and scala tympani (=PLS)
Grade 1	Displacement of RM, cochlear duct < scala vestibuli	ELS is enlarged and bulging into PLS	Irregular dilation and partial obstruction of the scala vestibuli, cochlear duct indirectly visible as nocular black cut-out of the scala vestibuli		**“X-mas tree”** with **“X-mas lights”**, where ELS is slightly enlarged and indirectly visible as a nodular black cut out (cp. Figure 1B).	**“X-mas tree”** (=enhancing scala vestibuli and scala tympani) with **“X-mas balls”** (=nodular enlargement non-enhancing scala media)
Grade 2	Displacement of RM, cochlear duct > scala vestibuli	Scala media is scalloping into the scala tympani, PLS has a semicircular appearance			**“X-mas tree”** with **“X-mas balls”**, where ELS is bulding into scala tympani whilst giving the PLS a semicircular appearance (cp. Figure 1C).	
Grade 3		A severely distended scala media causes a flattened appearance of the perilymph space	No scala vestibuli visible		**“X-mas tree”** with **“X-mas garlands”**, where ELS is distended and causes a flattened appearance of the PLS (cp. Figure 1D).	**“X-mas tree”** (=enhancing scala vestibuli and scala tympani) with **“X-mas garlands”** (=linear enlarged non-enhancing scala media)
**VESTIBULUM**						
*Slice of evaluation*	*Lowest slice of vestibulum L-SCC still visible*	*Same as Nakashima et al. (19)*	*Midmodiolar level*	*Axial slice through inferior part of vestibulum*	*Vestibulum inferior part; L-SCC still visible*	*Vestibulum inferior part*
Grade 0	AR <33.3%		AR <50%, sacculus and utriculus are distinguishable	SURI <1, no saccular abnormality	Sacculus < utriculus, otoliths still distinguishable (cp. Figure 1A).	AR <50%,sacculus < utriculus, otoliths are distinguishable
Grade 1	33.3% < AR <50%			SURI ≥1	Sacculus (sign should remain as is) utriculus, otoliths are still distinguishable (cp. Figure 1B).	Sacculus ≥ utriculus, otoliths are distinguishable
Grade 2	AR >50%		AR >50%, PLS remains visible with circular rim enhancement	No sacculus visible	Sacculus & utriculus are confluent, PLS rim visible (cp. Figure 1C).	Sacculus and utriculus are confluent, PLS remains visible with circular rim enhancement
Grade 3			No PLS visible		No otolith organs distinguishable, no PLS visible (cp. Figure 1D).	No PLS visible

*AR, area ratio; ELS, endolymphatic space; L-SCC, lateral semicircular canal; PLS, perilymphatic space; SURI, ratio ≥ 1 between the area of the sacculus and the area of the utriculus, RM, Reissner's membrane. The bold text highlights the main or most important characteristics*.

## Materials and Methods

### Setting and Institutional Review Board Approval

All data was acquired at the Interdisciplinary German Center for Vertigo and Balance Disorders (DSGZ) and the Department of Neurology of Munich University Hospital (LMU) between 2016 and 2019. Institutional Review Board approval was obtained before the initiation of the study (no. 641-15). All participants provided informed oral and written consent in accordance with the Declaration of Helsinki before inclusion in the study.

### Study Population

One hundred eight consecutive participants [75 patients with Meniere's disease (MD) and 33 vestibular healthy controls (HC)] underwent delayed intravenous gadolinium-enhanced magnetic resonance imaging (iMRI) for exclusion or verification of ELH. The diagnosis of Meniere's disease (MD) was based on the Classification Committee of the Bárány Society 2015 (33). HC were inpatients of the Department of Neurology without symptoms or underlying pathologies of the peripheral and central vestibular and auditory system that underwent MRI with a contrast agent as part of their diagnostic workup and agreed to undergo iMRI sequences after 4 h. HC underwent audio-vestibular testing to confirm the soundness of their peripheral end organs. The reasons for their admission to the clinic included movement disorders (*n* = 6), epilepsy (*n* = 5), optic neuritis (*n* = 4), trigeminal neuralgia (*n* = 4), headache (*n* = 4), idiopathic facial nerve palsy (*n* = 3), viral meningitis (*n* = 3), subdural hematoma (*n* = 2), spinal inflammatory lesion (*n* = 1), and decompensated esophoria (*n* = 1). The laterality quotient for right-handedness was assessed with the 10-item inventory of the Edinburgh test (34, 35). The inclusion criterion was age between 18 and 85 years. The exclusion criteria were other neurological or psychiatric disorders, as well as any MR-related contraindications (36), poor image quality, or missing MR sequences.

### Nomenclature

In the following, “ipsilateral” refers to the clinically leading side (or affected side) and “contralateral” to the opposite side (or non-affected side). In the case of patients presenting without a leading clinical side, a pseudorandom number generator [“Mersenne Twister” algorithm (37), uniform distribution] was used to generate a random number between 1 (=minimum value) and 9 (=maximum value). Even numbers meant “left side = ipsilateral side” and uneven numbers indicated “right = ipsilateral side.” “Vegetative symptoms” refers to nausea and/or vomiting due to the episodic vertigo attack. “Ear symptoms” includes attack-associated tinnitus, hearing loss, ear pressure, and/or ear pain both uni- and bilaterally that fit the criteria for MD. “Other ear symptoms” refers to non-MD ear symptoms.

### Measurement of the Auditory, Semicircular Canal, and Otolith Functions

Diagnostic workup included a thorough neurological workup (e.g., history-taking, clinical examination), neuro-orthoptic assessment [e.g., Frenzel glasses, fundus photography, and adjustments of the subjective visual vertical (SVV)], video-oculography (VOG) during caloric stimulation and head impulse test (HIT), as well as ocular (o) and cervical (c) vestibular evoked myogenic potentials (VEMPs) and pure tone audiometry (PTA).

A tilt of the SVV is a sensitive sign of a graviceptive vestibular tone imbalance. SVV was assessed with the subject sitting in an upright position in front of a half-spherical dome with the head fixed on a chin rest (38). A mean deviation of >2.5° from the true vertical was considered a pathological tilt of SVV.

The impairment of the vestibulo-ocular reflex (VOR) in higher frequencies was measured by HIT (39) using high-frame-rate VOG with EyeSeeCam [(40), EyeSeeTech, Munich, Germany]. A median gain during head impulses <0.6 (eye velocity in °/s divided by head velocity in °/s) was considered a pathological VOR (41). Furthermore, canal responsiveness in lower frequencies was assessed by caloric testing with VOG, which was performed for both ears with 30°C cold and 44°C warm water. Vestibular paresis was defined as >25% asymmetry between the right- and left-sided responses (42). The caloric asymmetry index (AI_C_) was calculated based on the slow-phase velocity of the caloric nystagmus: $AI_{C\;}\left[\%\right]\;=\frac{\left(R_{33{}^{\circ}C}+R_{44{}^{\circ}C}\right)-\left(L_{33{}^{\circ}C}+L_{44{}^{\circ}C}\right)}{\left(R_{33{}^{\circ}C}+R_{44{}^{\circ}C}\right)+\left(L_{33{}^{\circ}C}+L_{44{}^{\circ}C}\right)}\times \;100$.

Vestibular evoked myogenic potentials (VEMPs) are short-latency, mainly otolith-driven vestibular reflexes elicited by air-conducted sound (ACS), or bone-conducted vibration (BCV) and recorded from the inferior oblique eye muscle (ocular or oVEMPs) or the sternocleidomastoid muscle (cervical or cVEMPs). VEMPs were recorded with the Eclipse platform (Interacoustics, Middelfart, Denmark), as described previously (43, 44). Only those VEMP responses that were clearly discernible from background noise were included in the analysis. To avoid bias due to examiners, only the asymmetry index (AI_o/cV_) of VEMP amplitudes and latencies was analyzed in detail (45).

### Delayed Intravenous Gadolinium-Enhanced MRI of the Inner Ear

#### Data Acquisition

Four hours after intravenous injection of a standard dose (0.1–0.2 mmol/kg body weight, i.e., 0.1 −0.1 mmol/kg body weight) of Gadobutrol (Gadovist®, Bayer, Leverkusen, Germany), MR imaging (MRI) data were acquired in a whole-body 3 Tesla MRI scanner (Magnetom Skyra, Siemens Healthcare, Erlangen, Germany) with a 20-channel head coil. We used a 3D-FLAIR sequence to differentiate endolymph from perilymph and bone, and a CISS sequence to delineate the total inner ear fluid space from the surrounding bone. The T2-weighted, three-dimensional, fluid-attenuated inversion recovery sequence (3D-FLAIR) had the following parameters: TR 6,000 ms, TE 134 ms, TI 2,240 ms, FA 180°, FOV 160 × 160 mm^2^, 36 slices, base resolution 320, averages 1, acceleration factor of 2 using a parallel imaging technique with a generalized auto-calibrating partially parallel acquisition (GRAPPA) algorithm, slice thickness 0.5 mm, acquisition time 15:08 min. The high-resolution, strongly T2-weighted, 3D constructive interference steady state (CISS) sequence of the temporal bones was performed to evaluate the anatomy of the whole-fluid-filled labyrinthine spaces and had the following parameters: TR 1,000 ms, TE 133 ms, FA 100°, FOV 192 × 192 mm^2^, 56 slices, base resolution 384, averages 4, acceleration factor of 2 using GRAPPA algorithm, slice thickness of 0.5 mm and acquisition time 8:36 min. The presence of ELH was observed on the 3D-FLAIR images as enlarged negative-signal spaces inside the labyrinth, according to a previously reported method (18, 46).

#### Signal Quality Assessment

Signal quality was validated using signal-to-noise ratio (SNR) and signal-homogeneity (SH) in different regions of interest (ROIs). ROIs were labeled in the left and right inner ear within the “endolymph” and “perilymph” fluid, “cochlear basal turn,” as well as in the surrounding tissue or subject matter, such as the “petrous bone,” “cerebellum,” “medulla,” and “air.”

In detail, the endolymph ROI consisted of 0.6 mm^2^ circular 2D-selections of the left/right utricle. The perilymph ROIs consisted of multiple 0.6 mm^2^ circular 2-D selections in the perilymphatic space (PLS) on both sides and were spread within the inner ear to obtain a signal intensity map. Said selections were placed in the vestibulum, twice inside the basal cochlea turn, the apex cochleae, the horizontal semicircular canal (hSCC) as well as the posterior SCC (pSCC). ROIs in the surrounding tissue or subject matter (“petrous bone,” “cerebellum,” “medulla oblongata,” and “air”) consisted of 60.8 mm^2^ circular selections. Signal intensity extraction (mean, minimum, and maximum) was performed on axial slices of the FLAIR raw images via the “Analyze Regions” plugin of the “MorphoLibJ toolbox” (47) within ImageJ (48).

SNR was calculated in each ROI as $SNR\;\left(ROI\right)\;=\frac{S\left(ROI\right)}{std\left(air\right)\;}$, i.e., the fraction of mean signal intensity in an ROI S(ROI), and the standard deviation (STD) of the region labeled “air,” std(air). The label “air” was defined as “MRI signal measure of background variations in the signal devoid of fluid.” In other words, a region's SNR was calculated as a mean signal relative to the extent of the background variation.

The signal's statistical homogeneity was examined between ROIs for each group, and between groups for each ROI. SH was defined as the identical distribution of two samples except for shifts and scaling of the overall distribution. The median of each sample was removed and the interquartile range was scaled to the value of one. The two samples were then compared using *the minimum statistical energy* [*minEn*; (49)] and *the maximum mean discrepancy* [*MMD*; (50)], whilst adding 10,000 permutations with a threshold of maximally one failed test to reach statistical significance. Consequently, two samples were deemed to have different distributions if they diverged in shape, either due to kurtosis, skewness or the extent, and number of outliers. Note that no correction for multiple testing was applied in these tests in order to be more sensitive toward violations of SH, i.e., significant differences.

#### Semi-quantitative Grading of the Endolymphatic Space

**Figure 1:**
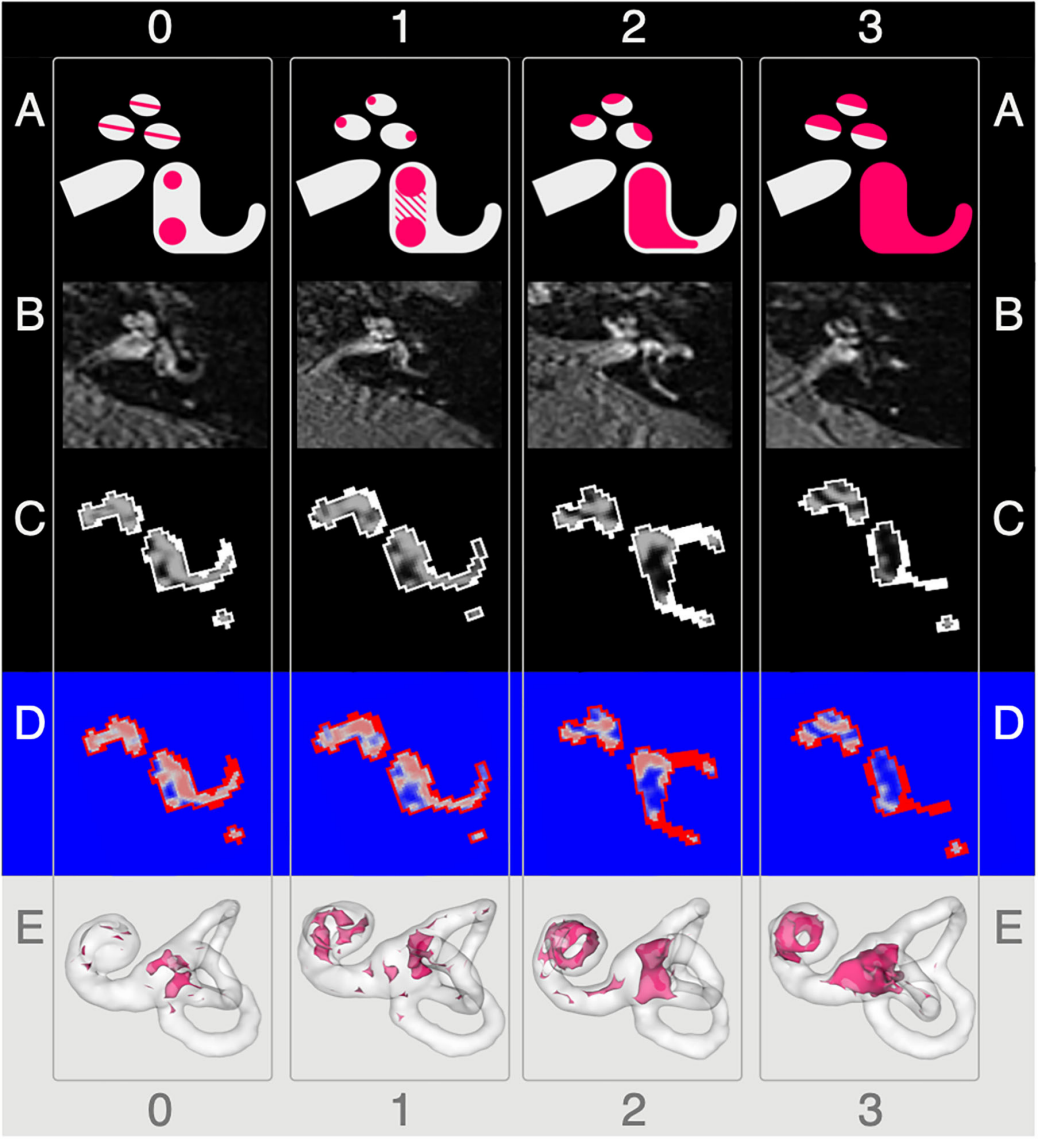
Semi-quantitative (SQ) grading used for the endolymphatic hydrops. The vertical columns show the different semi-quantitative (SQ) grades from 0 to 3 used [cf. Table 1; according to a classification first described in Kirsch et al. (9)]. The horizontal rows each give an overview of how each grade looks **(A)**, in FLAIR raw data **(B)**, after VOLT processing **(C)**, as used for 2D quantification **(D)**, and as used for 3D quantification **(E)**. A detailed description of each grade is given in the second paragraph of subsection ‘Semi-quantitative Grading of the Endolymphatic Space'.

Semi-quantitative (SQ) grading of the endolymphatic space (ELS) was performed independently by three experienced head and neck radiologists or neurologists (BE-W, VK, and JG) who were blinded to the clinical patient data. Rater statistical homogeneity was calculated just as the signal's statistical homogeneity. The ELS's characterization in the vestibulum and cochlea was based on criteria previously described (22) and can be viewed in Table 1 and is described in further detail in Figures 1A,B, grade 0–3.

**Figure 2:**
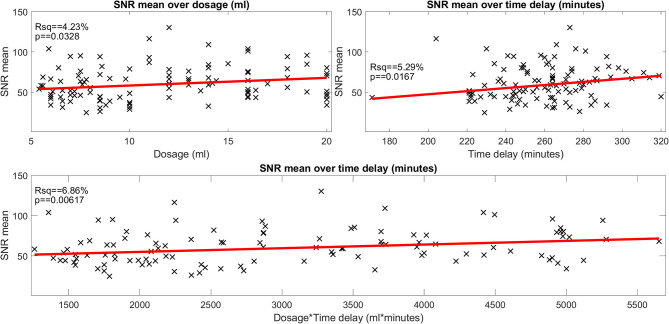
Influence of gadolinium (Gd) dosage and Gd time delay on the signal-to-noise ratio (SNR) (belonging to question i). The scatter plot of the signal-to-noise ratio (SNR) over the gadolinium (Gd) dosage (in ml, plot in the top left), of the SNR over the time delay (in minutes, plot in the top right) and of the SNR over the interaction of Gd dosage × time delay (ml × minutes, plot in the bottom). SNR data points are plotted as black crosses and the trend lines of the fitted model are plotted as red lines. The SNR depended significantly on dosage, time delay as well as the interaction of dosage × time delay. However, the effect size was very small (4.2, 5.3, and 6.8% of explained variance, respectively). The SNR displayed here “SNR mean” is the mean of the SNRs calculated for each region of interest, i.e., the two inside the basal cochlear turn (CBT), the apex cochleae (AC), the horizontal semicircular canal (hSCC) as well as the posterior SCC (pSCC). For each region, the SNR is the mean signal divided by the standard deviation of the region labeled “air.” The left and the right ear are averaged for each region, before regions are averaged to form “SNR mean”.

**Figure 3:**
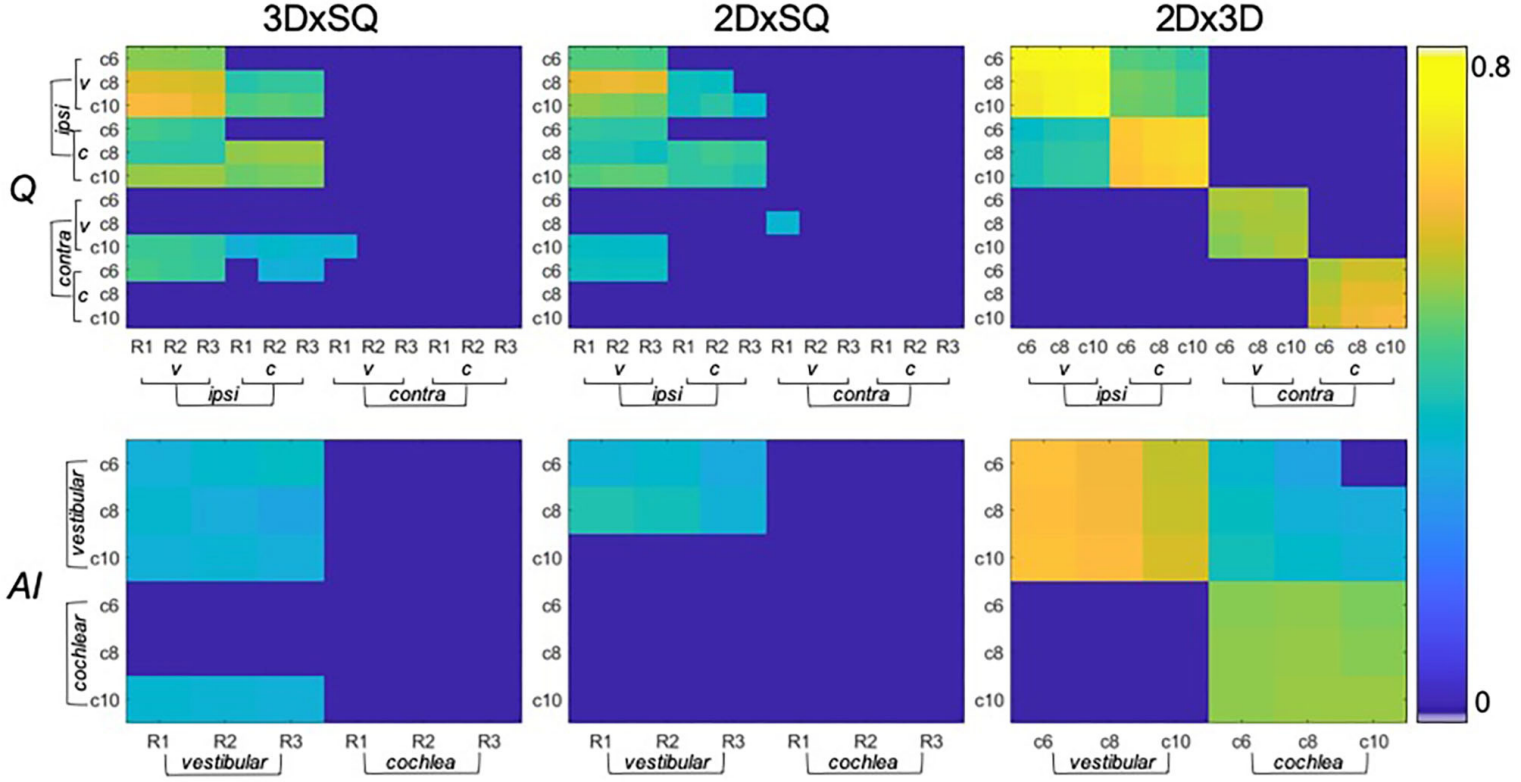
Significant correlations between endolymphatic hydrops (ELH) quantification methods (belonging to question ii). The top row shows the correlations between semi-quantitative (SQ) gradings (x-axis) and 2D- or 3D- quantification values (y-axis) for vestibulum (v) or cochlea (c), and for the ipsilateral (ipsi) or contralateral side (contra). In addition, SQ gradings are rater-specific (R1-R3), and 2D- or 3D- quantification values are cutoff-specific (c6, c8, and c10). The higher the significant results (*p* < 0.05 FWE-corrected) correlated, the more they are colored in yellow (thresholded to 0–0.8). Overall, SQ gradings and 2D- or 3D-quantification values correlated with the respective other method, although to a higher extent on the ipsilateral side (on the contralateral side in c10) and vestibular part of the inner ear. The bottom row shows the corresponding correlations between methods (SQ gradings and 2D- or 3D- quantification values) for the respective asymmetry-index (*AI*).

**Figure 4:**
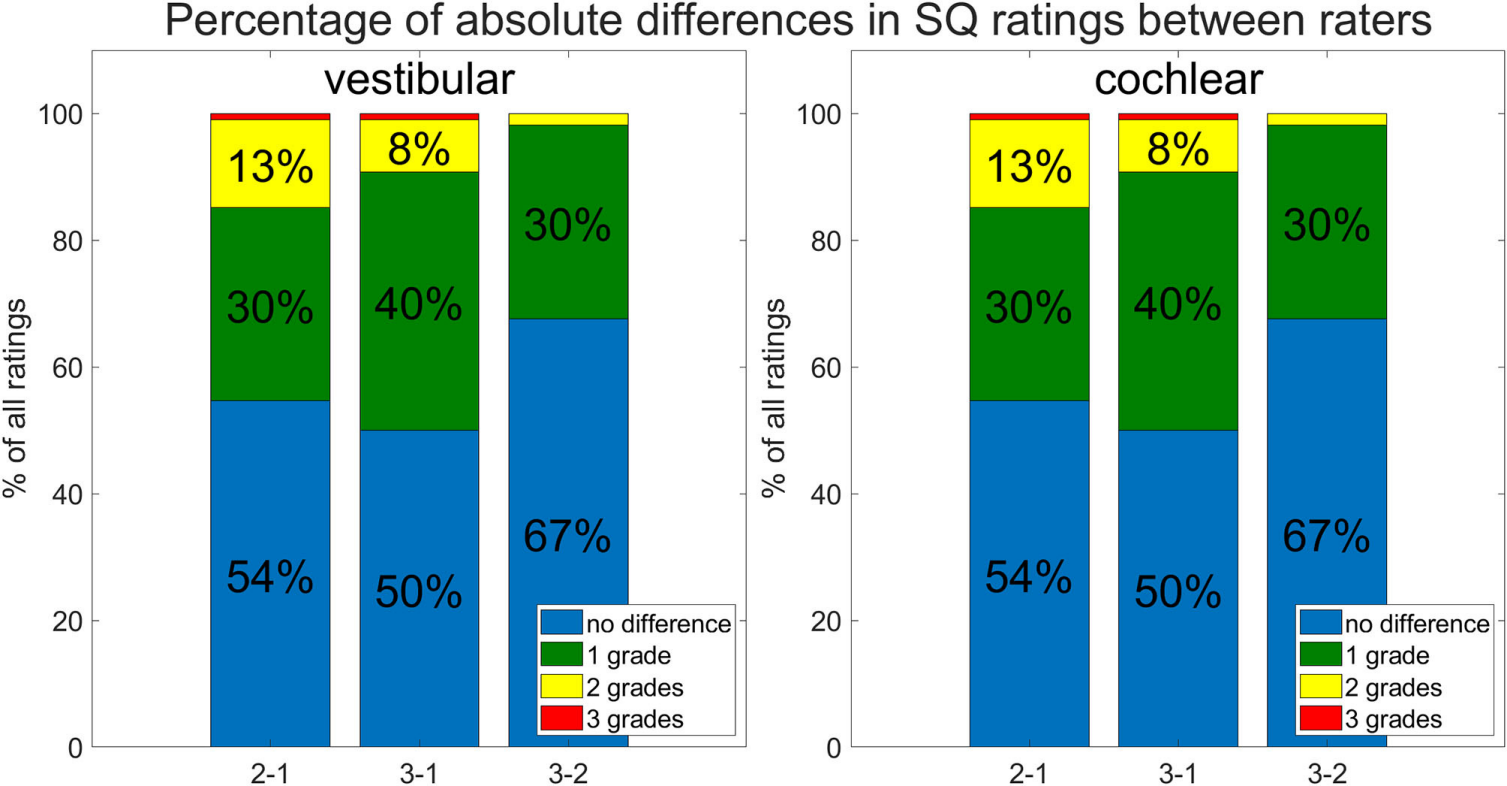
Inter-rater and -threshold between ELH quantification methods (belonging to question ii). Shown are differences between the three raters. The differences between raters are shown as percentages of the total number of subjects rated. Most grades between raters agree (no difference; in blue), and the next largest difference was by 1 grade (in green), the remaining differences between raters were mostly 2 grades (in yellow) and rarely 3 grades (in red) apart.

**Figure 5:**
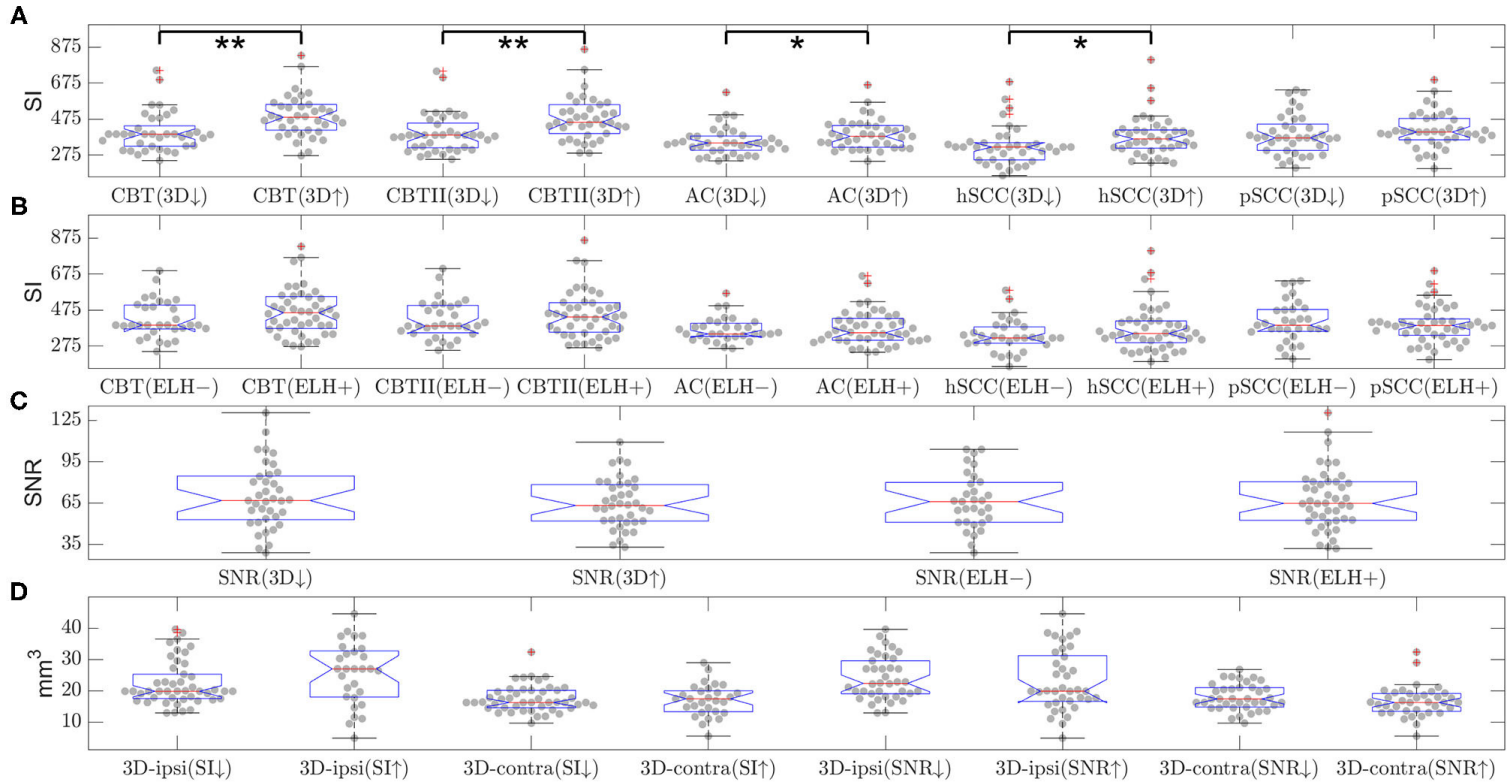
Influence of endolymphatic hydrops (ELH) on signal intensity (SI) or signal-to-noise ratio (SNR) and vice versa (belonging to question iii). The top two rows **(A,B)** show the signal intensity (SI) in various areas of the inner ear. The SI values are split by either **(A)** 3D-quantification values (below or above median, indicated by a downward-arrow or an upward-arrow) or **(B)** endolymphatic hydrops (ELH) being absent [“–”] or present [“+”]. Here, “ELH absence” is defined as the average semi-quantitative (SQ) rating being zero and “ELH presence” is defined as the average rating being non-zero. Significant differences are indicated by black lines and p-values for the group differences evaluated with two-sample *t*-test or rank-sum test (**p < 0.001 and *p < 0.05). The third row **(C)** shows the mean SNR split by 3D-quantification values (below or above median; indicated by a downward-arrow or an upward-arrow), as well as the mean SNR split by ELH being absent [“–”] or present [“+”], where presence is defined as ELH being non-zero and absence as ELH rating being zero. The group differences were evaluated with the two-sample *t*-test and the rank-sum test; neither of these was significant. The fourth row **(D)** shows the 3D-quantification values split by mean SI values (below or above median; indicated by a downward-arrow or an upward-arrow), as well as the 3D-quantification values split by SNR values (below or above median; indicated by a downward-arrow or an upward-arrow). The group differences were evaluated with the two-sample *t*-test and the rank-sum test; neither of these was significant.

**Figure 6:**
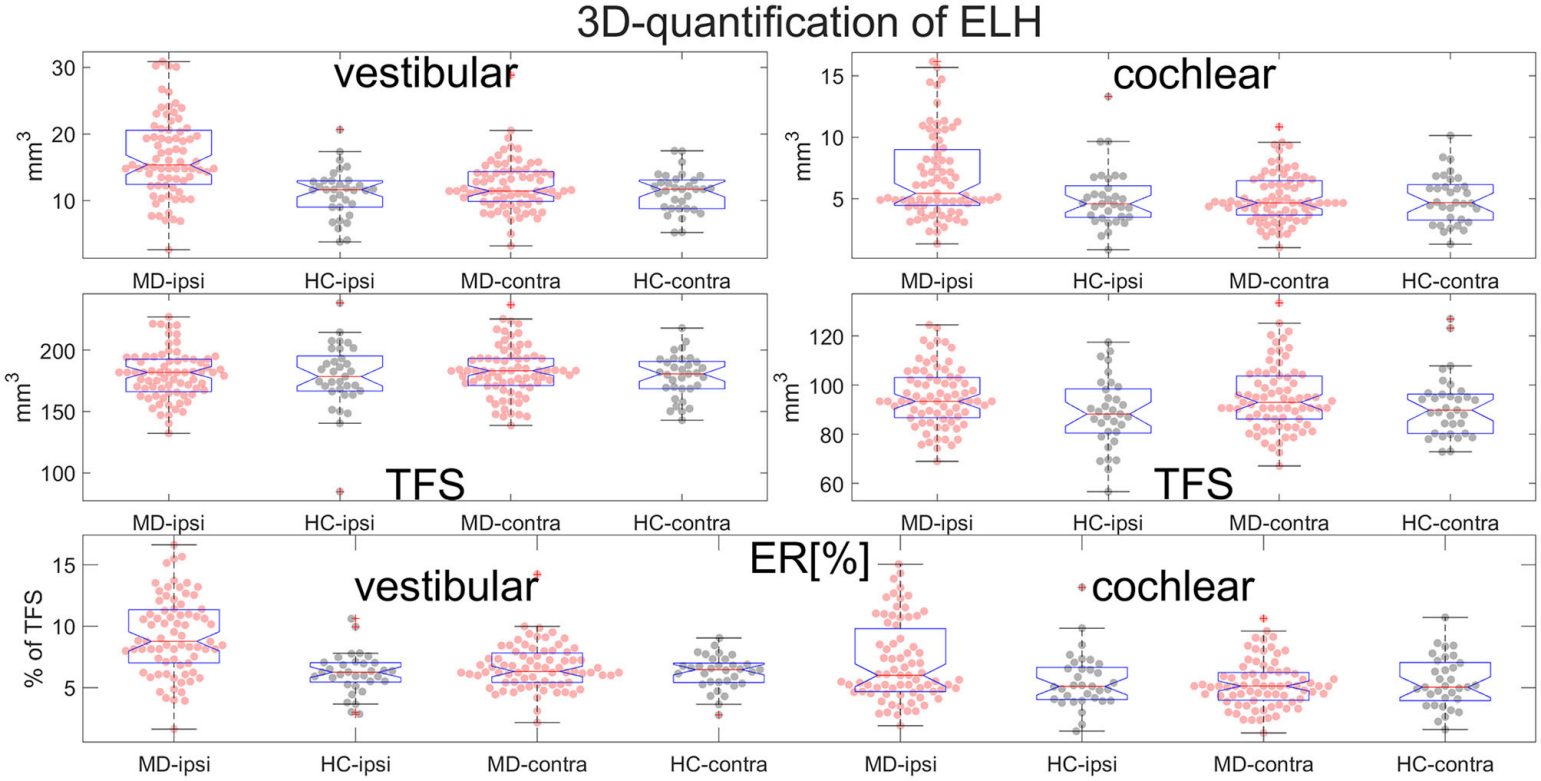
Normalization and group differences for 3D-quantification of endolymphatic hydrops (ELH). 3D-quantification values of endolymphatic hydrops (ELH) in the vestibular and cochlear part of the inner ear [in mm^3^] (top). 3D-quantification values of the total fluid space (TFS) of the vestibular and cochlear part of the inner ear [in mm^3^] (middle row) that were used for normalizing the data of each individual to ELS ratio (ER) [%], the percentage of the TFS occupied by the ELH (bottom row).

**Figure 7:**
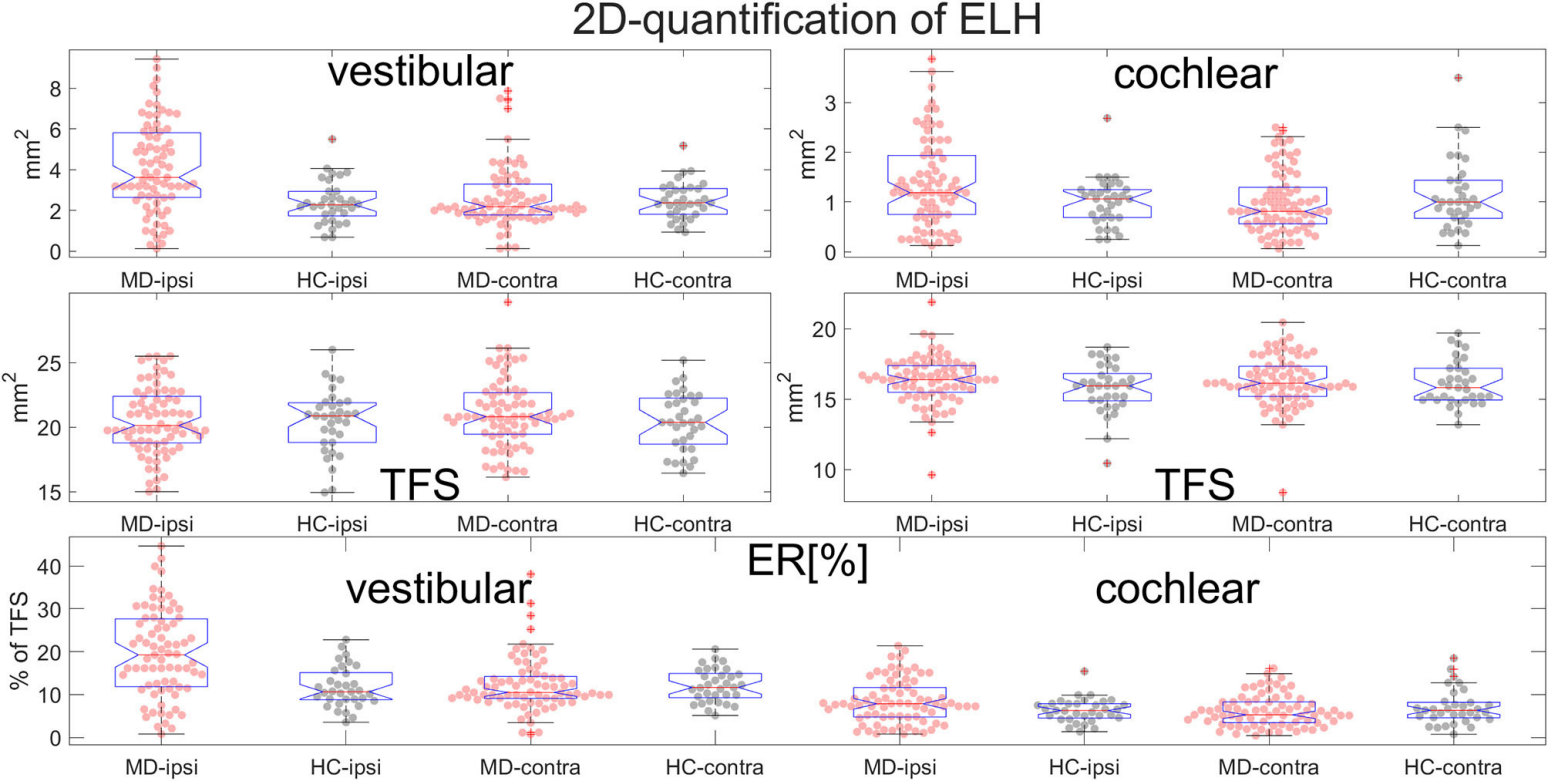
Normalization and group differences for 2D-quantification of endolymphatic hydrops (ELH). 2D-quantification values of endolymphatic hydrops (ELH) in the vestibular and cochlear part of the inner ear, in [mm^2^] (top row). 2D-quantification values of the total fluid surface (TFS) of the vestibular and cochlear part of the inner ear, in [mm^2^] (middle row) that were used for normalizing the data of each individual to ELS ratio (ER) [%], the percentage of the TFS occupied by the ELH (bottom row).

**Figure 8:**
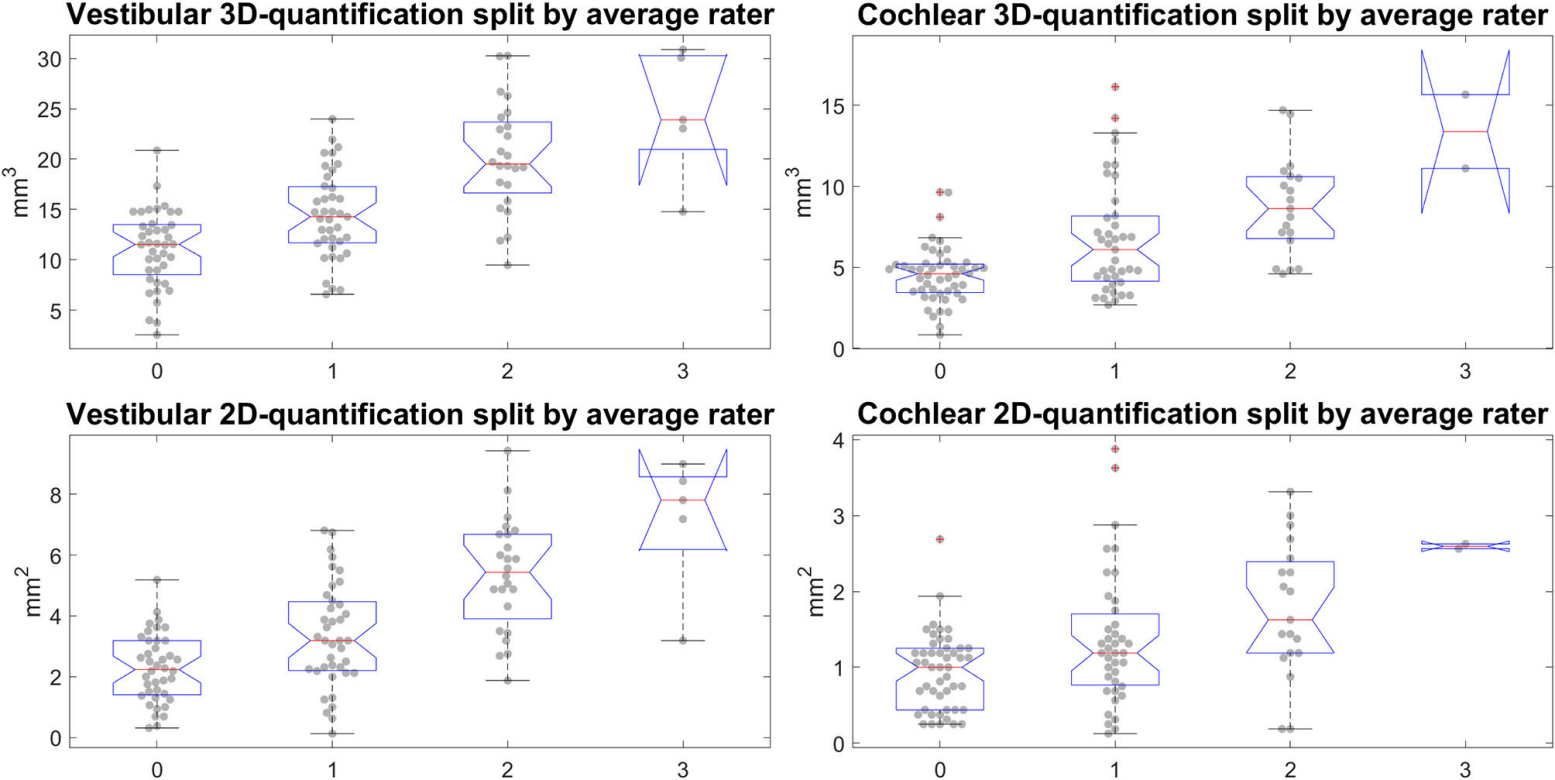
Semi-quantitative (SQ) grade-specific 2D- and 3D-quantification values. These four plots show the quantitative endolymphatic hydrops (ELH) measures (3D-quantification in the top row and 2D-quantification in the bottom row) split up along the semi-quantitative visual grading steps (from grade 0 to grade 3 in steps of 1). This means each boxplot with data points overlaid shows the distribution of quantitative measures that correspond to the respective grading. The grading here is of the average over the three raters, rounded to the nearest integer to preserve gradings going from 0 to 3 in steps of 1. The top row shows the distributions for the 3D-quantification of ELH for vestibular and cochlear parts of the inner ear, left and right, respectively, while the bottom row shows the distributions for the 2D-quantification of ELH for vestibular and cochlear parts of the inner ear.

The characterization describes a 4-point grading for the cochlear and vestibular ELH. The cochlear grading is done on the midmodiolar level (19) and the vestibular grading on the inferior part of the vestibulum, where the left semicircular canal (L-SCC) is still visible (19). The cochlear grading can be thought of as a fusion of previously described grading suggestions (21–24). Grade 0 (*no vestibular ELH*) can be reduced to “X-mas tree built from circles that are divided by “very thin, clear, hypointense lines” [cp. also Figure 1 of (23), Figure 1A in (21)] that represent the non-enhanced ELS (scala media) between the enhanced PLS (scala vestibuli and tympani). Grade 1 *(mild cochlear ELH)* can be reduced to “X-mas tree with lights,” where the ELS is slightly enlarged and indirectly visible as a nodular black cut out of the scala vestibuli [cp. further Figure 2 in (24), Figure 1B in (21)]. Grade 2 (*marked cochlear ELH*) can be reduced to “X-mas tree with X-mas balls,” where the ELS is bulging into the scala tympani whilst giving the PLS a semicircular appearance [cp. Figure 3 in (24)]. Grade 3 (*severe cochlear ELH*) can be reduced to “X-mas tree with garlands,” where the severely distended ELS is causes a flattened appearance of the PLS [cp. also Figure 4 in (24), Figure 5A in (21), Figure 1C in (20)]. The vestibular grading is a fusion of previously described grading suggestions (21–23). Grade 0 (*no vestibular ELH*) can be reduced to “sacculus < utriculus,” where the otolith organs are distinguishable and the sacculus is smaller than the utriculus [cp. also Figure 2A in (21), Figure 6 in (23)]. Grade 1 (*mild vestibular ELH*) can be reduced to “sacculus≥utriculus,” where the sacculus is as large or larger than the utriculus [cp. also Figure 2B in (21), Figure 9 in (23)]. Grade 2 (*marked vestibular ELH*) can be reduced to “sacculus and utriculus are confluent,” where the otoliths organs are no longer distinguishable with a surrounding PLS rim [cp. also Figure 2C in (21), Figure 7 in (23)]. Grade 3 (*severe vestibular ELH*) can be reduced to “otolith organs not distinguishable” with no PLS visible [cp. also Figure 2D in (21), Figure 8 in (23)].

#### 2D- and 3D-Quantification of the Endolymphatic Space

Segmentation of the total fluid space (TFS) was based on a recently proposed (Ahmadi et al., under review) and pre-trained volumetric deep convolutional neural network (CNN) with V-net architecture (51) that was deployed via the TOMAAT module (52) in 3D–Slicer toolbox [version 4.11 (53)]. ELS and PLS were differentiated within the TFS using Volumetric Local Thresholding [VOLT; (31)] using ImageJ Fiji (48) with the “Fuzzy and artificial neural networks image processing toolbox” (54) and the “MorphoLibJ Toolbox” (47).

The resulting 3D volume can be regarded as a probabilistic map of the inner ear, which includes the classification into its two different compartments (ELS and PLS). The final classification strongly depends on the chosen cutoff. Based on empirical observations (31), 2D- and 3D-quantifications were examined at three cutoff variations (c6, c8, and c10). Each cutoff matches a percentage of positive classifications. For example, cutoff 6 (c6) corresponds to 79.2%, cutoff 8 (c8) to 70.8%, and cutoff 10 (c10) to 62.5% classifications into endolymphatic space. Examples of the pipeline outputs can be viewed in Figure 1C.

2D-quantification was done on axial slices of the VOLT volume. The mid-modiolar level was chosen for the cochlea and the inferior part of the vestibulum where the lateral semicircular canal (L-SCC) is still visible was selected for the vestibulum. However, the majority of volumes allowed for both a cochlear and vestibular measurement on the same slice. Easier visual selection was enabled by a look-up-table (LUT, “phase”) included in ImageJ that was applied to the VOLT volumes. An example can be seen in Figure 1D. Areas were then measured using the “Analyze Regions” plugin which is part of the “MorpholibJ Toolbox” (47).

3D-quantification was done on the VOLT volume that included the entire inner ear. The cochlear volume was cropped using a cylindrical volumetric selection and applied to the VOLT volumes. The volume of the vestibulum including otolith organs and semicircular canals arose from subtracting the cochlear volume from the inner ear VOLT volume. Measurements were performed using the “Analyze Regions (3D)” plugin of the “MorpholibJ Toolbox” (47). A visualization can be seen in Figure 1E.

#### Parameters Derived From Endolymphatic Space Measures

The ELS ratio, $ER\;\left[\%\right]=\frac{ELS}{TFS}\times 100$, was calculated for 2D- and 3D-quantification of the ELS analogous to the area ratio (AR) in previous classification conventions (19–21). ER indicates the relative size of the ELH to the TFS and as such is independent of the absolute size which might differ between subjects (for example due to body size).

ELS symmetry between both inner ears was assessed via the ratio of ELS side differences *Diff* − *ER* [%] = *ERi* − *ERc*, where *ERi* and *ERc*are the respective ipsilateral and contralateral ELS ratios in percent relative to the TFS. Another parameter was the asymmetry index, $AI\;\left[\%\right]=\frac{\left(ELSi\;ELSc\right)}{\left(ELSi\;+ELSc\right)}\times 100$, where *ELS*_*i*_ is the semi-, 2D- or 3D-quantification of the ipsilateral ELS and *ELS*_*c*_ of the contralateral ELS. The asymmetry index can be interpreted as a normalized difference and as such is also independent of the individual TFS.

Areas and volumes were normalized according to their TFS,

if _*c/v/a*_$TFS_{e2D/3D}^{c8}$ > _*c/v/a*_$TFS_{mean\;2D/3D}^{c8}$ + 2.5 × *std* (*TFS*), where “*e*” is the individual value and “*mean*” is the mean of the respective group (HC or MD). For an overview of TFS, see Figure 6.

### Statistics and Validation Parameters

All statistics were implemented with self-written scripts in MATLAB version 7.19.0 (R2019b) using the “Statistics and Machine Learning” toolbox provided with MATLAB (Natick, Massachusetts: the MathWorks Inc.). ELS quantification measures were validated and compared using parameters describing different characteristics on different levels (i.e., between groups, ELS analysis methods, and diagnostic methods) and between different entities (i.e., inter-rater, inter-threshold, and inter-ROI). Parameters considered the ordering of subjects between samples (concordance), Spearman correlations between samples (rank-correlation), the form of the distribution of samples via “*minimum statistical energy*” and “*maximum mean discrepancy*” (statistical homogeneity), and covariance between samples via ANCOVA (analysis of covariance). All statistical tests used multiple comparison correction, if multiple tests (e.g., more than two regions or two thresholds) were compared independently with each other. The FWE level was set at *p* = 0.05/N with N being the number of tests (e.g., regions, thresholds), i.e., Bonferroni correction.

#### Influence of Gd Dosage, Gd Time Delay on SNR and via SNR on SQ Grading, 2D- or 3D-Quantitatification

The influence of Gd dosage and time delay (from Gd injection to MR measurement) on the SNR and signal intensity (SI), as well as SNR, Gd dosage and time delay on SQ grading, 2D- or 3D- quantification measures was evaluated using ANCOVA modeling. The model included interaction of the group with each individual variable as well as the interaction of group, dosage and time delay variables. Additionally, covariates of no interest, such as age and BMI, were included. In other words, we checked whether SNR, Gd dosage, and Gd time delay each had an influence on the ELH measure in question, as well as the interaction of Gd dosage and Gd time delay, allowing for the possibility that the relationship might be different for each group.

#### Interrelations Between SQ Gradings and 2D- or 3D Quantification

##### Statistical Homogeneity

Statistical homogeneity between SQ grading, and 2D- or 3D- quantification methods between groups was, in principle, calculated in the same way as the signal statistical homogeneity (cf. signal quality validation). First, the median of each group was removed and the interquartile range was scaled to the value of one. The two groups were then compared using *minEn* and *MMD test statistics* whilst using 10,000 permutations between groups. Any instance of a random permutation with a higher test-statistic than the unpermuted groups was considered a failure. The groups were deemed statistically homogeneous if at most one test failed, otherwise the groups were deemed inhomogeneous, as they could be distinguished based solely on their distribution shape (kurtosis and skewness or the extent and number of outliers). Note that no correction for multiple comparisons was performed here in order to be more sensitive to violations of SH, i.e., significant differences.

##### Rater Repeatability and Reliability

Repeatability and reliability of the three different raters for SQ grading, as well as of the three different thresholds (c6, c8, and c10) for 2D- or 3D-quantification were measured using rank-based correlations and Kendall's W measure for concordance (55). This assessment shows whether the ordering of subjects between raters is similar and therefore can be assumed to be repeatable over the raters. Furthermore, we compared ratings by subtracting the SQ grading scores between raters to see if the extent of differences in rating values differed. Correction for multiple comparisons, i.e., multiple tests was done over data types (SQ, 2D and 3D), therefore *p*_(FWE)_ = 0.05 was set to *p* = 0.05/N with *N* = 3 for the three data types.

##### Interrelations Between SQ Grading, 2D- and 3D-Quantification

Interrelations between SQ grading, 2D- and 3D-quantification were examined via Spearman, i.e., rank-based correlations. Significant rank correlations indicated that the ordering of subjects was very similar or concordant across these measures. Rank-correlation was used so that linear as well as non-linear relationships could be examined and the gradings (ordinal measures) could be related to the quantitative measures. Correction for multiple comparisons *p*_(FWE)_ = 0.05/N, i.e., Bonferroni correction, was done over all pairs of correlations in each correlation matrix, i.e., for SQ- × -2D quantification and SQ- × -3D quantification *N* = 12- × 12 = 144 and for the correlation of asymmetry indices *N* = 6- × 6 = 36.

##### Influence of Thresholds on Quantitative Measures

The influence of VOLT thresholds (c6, c8, and c10) on group differences was assessed using general linear model (GLM) based two-sample *t*-tests (including age as a covariate of no interest). The resulting slopes for the effects of thresholds and their standard errors were used to calculate *t*-statistic values for each group comparison at each threshold. Furthermore, a slope difference test (56) was used to check if group differences depended on the VOLT thresholds. A slope difference test compares differences in slopes with standard errors for the group differences across thresholds to determine if group differences depended on the cutoff-threshold. Correction for multiple comparisons *p*_(FWE)_ = 0.05/N, i.e., Bonferroni correction, was done for three tests of between threshold comparisons resulting from three thresholds (c6,c8,c10), i.e., c6-vs-c8, c6-vs-c10, and c8-vs-c10, and therefore *N* = 3.

##### Covariance of Clinical Measures and iMRI

Clinical (e.g., disease duration, number of attacks) and diagnostic measures (e.g., HIT, calorics, and VEMPs), as well as parameters derived from ELS measures in SQ gradings and 2D- and 3-D quantifications (*ER, Diff-ER*, and *AI*) were included in an analysis of covariance (ANCOVA). An overview of clinical symptoms and diagnostic measures can be viewed in Table 2. Furthermore, the analysis accounted for categorical variables, such as symptoms like headache, and continuous covariates, such as body mass index (BMI) and the age of the patients. For detection of diverging trends between MD or HC, parameters derived from ELS measures were allowed interactions with the group. That means each group was allowed to have a different trend in the model. We used Bonferroni-correction for the *post-hoc* assessment of the individual factors in the ANCOVA.

**Table 2:** Clinical syndrome and diagnostic characteristics.

		**MD**		**HC**
	**All**	**Definite**	**Probable**	**All**
	***n* = 75**	***n* = 35**	***n* = 40**	***n* = 33**
Age [in years]	55.2 ± 14.9	54.8 ± 14.1	55.6 ± 15.8	42.1 ± 18.9
Age range	22–81	27–77	22–81	20–84
Gender	36 females	14 females	22 females	19 females
Handedness	97% RH, 3% LH	100% RH	93% RH, 7% LH	97% RH, 3% LH
**(A) Clinical syndrome**				
Type of vertigo	73% Ro, 25% Sw, 2% Lh	83% Ro, 14% Sw, 3% Lh	64% Ro, 33% Sw, 3% Lh	–
Duration of illness [in months]	56.8 ± 88.7	49.0 ± 84.9	63.5 ± 92.4	–
Number of attacks altogether	48.7 ± 56.6	47.2 ± 49.1	49.8 ± 62.7	–
Number of attacks in the last 3 months	15.0 ± 37.8	18.5 ± 52.4	12.4 ± 21.2	–
Duration of attacks [in hours]	5.0 ± 6.9 (0.5–24)	3.2 ± 3.0 (0.5–12)	6.7 ± 8.7 (0.5–24)	–
Time since last attack [in days]	35.1 ± 44.7 (1–180)	36.1 ± 43.4 (1–180)	33.9 ± 47.0 (1–180)	–
Nausea, Vomiting	86.7%	97.1%	77.5%	–
VM-Headache	1.3%	0%	2.5%	–
Sensitivity to light or noise	9.7%	8.6%	10.0%	–
Focal neurological deficits	8.0%	0%	15%	–
History of migraine	1.7%	3.1%	0%	–
Family history of migraine	5.1%	0%	11.5%	–
Other-Headache	22.7%	28.6%	17.5%	–
MD-Ear-symptoms	84.0%	94.3%	75.0%	–
MD-bilateral	25.3%	31.4%	20.0%	–
MD-ipsilateral	81.3%	94.3%	70.0%	–
MD-contralateral	8.0%	11.4%	5.0%	–
Other-Ear-symptoms	8.0%	2.9%	12.5%	–
Other-bilateral	8.0%	2.9%	12.5%	–
Other-ipsilateral	9.3%	0%	15%	–
Other-contralateral	0%	0%	0%	–
**(B) Diagnostic characteristics**				
CEMD	9.3%	2.9%	15%	0%
PEMD ipsilateral	53.3%	60.0%	47.5%	0%
SVV ipsilateral, pathologic	28%	34.3%	22.5%	0%
Caloric ipsilateral, pathologic	83.6%	94.3%	73.7%	0%
Caloric contralateral, pathologic	0%	0%	0%	0%
Caloric bilateral, pathologic	1.4%	0%	2.6%	0%
Caloric ipsilateral [°/s]	7.6 ± 6.9 (0.8–40.6)	7.8 ± 7.8 (0.9–40.3)	7.5 ± 6.1 (0.8–34.4)	13.8 ± 4.5 (3.8–24.2)
Caloric contralateral [°/s]	12.8 ± 8.4 (1.6–55.0)	13.8 ± 9.7 (1.6–55.0)	11.9 ± 7.0 (2.7–32.5)	19 ± 11 (4.8–50.1)
Caloric Asymmetry-Index [%]	34.8 ± 22.7 (0.7–90.6)	36.4 ± 23.3 (0.9–40.6)	33.3 ± 22.2 (0.8–90.6)	0.2 ± 19.8 (0.8–48.3)
HIT ipsilateral, pathologic	48.4%	51.6%	45.5%	0%
HIT bilateral, pathologic	6.3%	6.5%	6.1%	0%
HIT ipsilateral [gain at 60ms]	0.8 ± 0.2 (0.2–1.1)	0.7 ± 0.2 (0.2–1.02)	0.8 ± 0.2 (0.4–1.1)	1 ± 0.1 (0.8–1.1)
HIT contralateral [gain at 60 ms]	0.8 ± 0.2 (0.4–1.1)	0.8 ± 0.2 (0.4–1.01)	0.9 ± 0.1 (0.6–1.09)	0.9 ± 0.1 (0.8–1.0)
Audio MD-typical ipsilateral	76.2%	93.3%	60.6%	0%
Audio MD-atypical ipsilateral	3.2%	0%	6.1%	0%
Audio Presbyacusis-typical	0%	0%	0%	0%
Audio low-frequency ipsilateral [dB]	41.3 ± 23.1 (8.0–110.0)	49.9 ± 20.3 (10.0–110.0)	33.7 ± 22.9 (8.0–92.0)	20.0 ± 3 (8.0–35.0)
Audio low-frequency contralateral [dB]	21.1 ± 14.4 (7.0–77.0)	20.3 ± 11.6 (7.0–63.0)	20.3 ± 11.6 (7.0–63.0)	18 ± 6 (15–33.0)

*CEMD, central eye movement disorder; Lh, light-headedness; PEMD, peripheral eye movement disorder; Ro, rotational vertigo; Sw, swaying vertigo*.

#### Influence of ELH Presence on SNR and SI

The influence of the presence and extent of ELH on SNR and SI was examined with two approaches. First, SNR and SI data were investigated using classifications derived from SQ grades and 3D-quantification measures. The SQ grades were used to distinguish between “no ELH” and “ELH present,” while the 3D-quantification was used to distinguish between “low/small ELH” and “high/large ELH.” For the classification using SQ grades, all grades equal to zero (SQ grade = = 0) were allocated to “no ELH” and the rest to “definite ELH present.” For the classification using 3D-quantification, data values below the median were in the “low ELH” class and data values above the median in the “high ELH” class. The SNR and SI data were analyzed using two-sample *t*-tests and Wilcoxon rank-sum tests for differences from these classifications. The two tests (i.e., a parametric and non-parametric test), were used to ensure that any of the significant differences found were not purely dependent on the assumed distribution. Correction for multiple comparisons *p*_(FWE)_ = 0.05/N, i.e., Bonferroni correction, was done for five tests between regions (split by ELH) comparisons, i.e., *N* = 5 (see Figures 5A,B).

Then, the inverse question was asked. This time SQ and 3D-quantification values were compared following SI or SNR value classification and then analyzed accordingly for differences with two-sample *t*-tests and Wilcoxon rank-sum tests. For both SI and SNR classification, “*low SI or SNR class*” was defined by their values below the respective median, and “*high SI or SNR class*” by their values above the respective median. Correction for multiple comparisons *p*_(FWE)_ = 0.05/N, i.e., Bonferroni correction, was done separately for the test between SNR (split by ELH 3D-quantification) and 3D-quantification (split by SI). The number of tests for the SNR comparison was *N* = 2, and the number of tests for ELH 3D-quantification was *N* = 4 (see Figures 5C,D).

## Results

### Descriptive Statistics

Seventy-five MD patients (35 females; aged 22–81 years, mean age 56.6 ± 14.9 years; 97% RH) and 33 HC participants (20 females; aged 20–84 years, mean age 42.1 ± 18.9 years; 94% RH) were included in the study. An overview of the most important clinical features in MD compared to HC can be seen in Table 2. An overview of the ELS grading for HC and MD can be viewed in Table 3.

**Table 3:** Semi-quantitative (SQ) grading, 2D- and 3D-quantification of the ELS.

								**MD**										**HC**				
			**All**					**Definite**					**Probable**					**All**				
			**(*****n*** **=** **75)**					**(*****n*** **=** **35)**					**(*****n*** **=** **40)**					**(*****n*** **=** **33)**				
**(A) EH presence [%]**																						
			**R1**	**R2**	**R3**	**R1-3**		**R1**	**R2**	**R3**	**R1-3**		**R1**	**R2**	**R3**	**R1-3**		**R1**	**R2**	**R3**	**R1-3**	
Ipsilateral	ELS		80%	69%	85%	78%		86%	83%	91%	87%		75%	57%	80%	71%		9%	39%	27%	25%	
	cELS		65%	60%	67%	64%		74%	74%	74%	74%		57%	48%	60%	55%		6%	21%	9%	12%	
	vELS		72%	61%	69%	68%		74%	74%	80%	76%		70%	50%	60%	60%		9%	33%	27%	23%	
Contralateral	ELS		73%	44%	72%	63%		77%	37%	77%	64%		70%	50%	68%	63%		21%	27%	27%	25%	
	cELS		59%	32%	40%	44%		57%	23%	37%	39%		60%	40%	43%	48%		15%	21%	9%	15%	
	vELS		61%	36%	56%	51%		63%	34%	71%	56%		60%	38%	43%	47%		21%	24%	24%	23%	
**(B) SQ grading**																						
**Grades**			**0**	**1**	**2**	**3**		**0**	**1**	**2**	**3**		**0**	**1**	**2**	**3**		**0**	**1**	**2**	**3**	
Ipsilateral	ELS	R1-3	15%	15%	13%	5%		9%	14%	17%	3%		20%	15%	10%	8%		73%	9%	0%	0%	
	cELS	R1-3	33%	35%	27%	5%		26%	34%	37%	3%		40%	35%	18%	8%		91%	9%	0%	0%	
	vELS	R1-3	31%	31%	24%	15%		20%	26%	40%	14%		40%	35%	10%	15%		73%	27%	0%	0%	
Contralateral	ELS	R1-3	28%	20%	8%	1%		23%	23%	9%	3%		33%	18%	8%	0%		73%	6%	0%	0%	
	cELS	R1-3	60%	28%	11%	1%		63%	23%	11%	3%		57%	33%	10%	0%		91%	9%	0%	0%	
	vELS	R1-3	44%	39%	15%	3%		29%	51%	17%	3%		57%	28%	13%	3%		76%	24%	0%	0%	
Percentage		R1-3	**Min**	**25%**	**50%**	**75%**	**Max**	**Min**	**25%**	**50%**	**75%**	**Max**	**Min**	**25%**	**50%**	**75%**	**Max**	**Min**	**25%**	**50%**	**75%**	**Max**
AI [%]	ELS	R1-3	0	0	33	100	100	0	20	33	92	100	0	0	43	100	100	0	0	0	0	100
	cELS	R1-3	0	0	50	100	100	0	0	100	100	100	0	0	33	100	100	0	0	0	0	100
	vELS	R1-3	0	0	33	100	100	0	0	33	88	100	0	0	27	100	100	0	0	0	0	100
**(C) 2D-quantification**																						
			**Mean**	**Std**	**25%**	**50%**	**75%**	**Mean**	**Std**	**25%**	**50%**	**75%**	**Mean**	**Std**	**25%**	**50%**	**75%**	**Mean**	**Std**	**25%**	**50%**	**75%**
Ipsilateral	ELS	c8	5.5	2.7	3.4	5.2	7.4	6.3	2.8	4.5	6.2	8.5	4.7	2.4	3.1	4.4	6.5	3.4	1.3	2.5	3.3	4
[mm^2^]	cELS	c8	1.4	0.9	0.8	1.2	1.9	1.6	0.9	0.9	1.4	2.3	1.2	0.9	0.5	1.2	1.5	1	0.5	0.7	1.1	1.3
	vELS	c8	4.1	2.2	2.6	3.6	5.8	4.7	2.4	3.3	4.9	6.2	3.5	1.9	2.3	3.2	4.8	2.4	1.1	1.7	2.3	2.9
Contralateral	ELS	c8	3.7	1.8	2.6	3.3	4.5	3.9	1.9	2.7	3.5	4.8	3.5	1.7	2.6	3.3	4	3.6	1.2	2.7	3.8	4.5
[mm^2^]	cELS	c8	1	0.6	0.6	0.8	1.3	1.1	0.7	0.4	1	1.8	0.9	0.5	0.6	0.8	1.2	1.1	0.7	0.7	1	1.4
	vELS	c8	2.6	1.5	1.8	2.2	3.3	2.8	1.5	2	2.4	3.6	2.5	1.6	1.7	2.1	2.7	2.5	0.9	1.8	2.4	3.1
AI [%]	ELS	c8	16.1	28.2	0.2	14.8	34.6	20.7	27.2	5.1	22.7	45.8	12.1	28.7	0.7	14.7	31.1	4.0	20.4	17.3	7.02	12.7
	cELS	c8	13.2	40.4	5.6	17.9	34.2	17.3	41.4	5	14.3	46.1	9.6	39.8	6.6	18.8	33.3	2.5	32.5	27.0	0	22.9
	vELS	c8	17.9	33.8	0.9	21.4	38.6	20.5	34.8	0.9	28.6	45.7	15.7	33.2	0.6	21.4	30.0	3.2	24.6	16.5	1.5	9.5
ER [%]	ELS	c8	14.71	6.97	10.09	13.7	20.44	16.61	6.85	11.14	16.67	21.52	13.05	6.73	7.24	11.99	17.54	9.33	3.22	7.18	8.66	11.04
Ipsilateral	cELS	c8	8.4	5.1	4.8	7.9	11.6	9.4	5.1	5.5	8.6	13.2	7.6	5	3.3	7.3	9.7	6.3	2.8	4.5	6.3	7.9
	vELS	c8	19.7	10.3	11.9	19.2	27.7	22.3	10.7	13.8	23.1	30.3	17.4	9.5	11.1	16.1	23.8	11.7	4.8	8.8	10.7	15.1
ER [%]	ELS	c8	9.7	4.1	7.3	8.9	11.6	10.4	4.5	7.3	9.6	13	9.1	3.7	7.3	8.8	9.9	9.9	2.7	7.6	10.3	12.4
Contralateral	cELS	c8	6.2	3.7	3.4	5.3	8.3	6.8	4.2	2.7	6.3	10.8	5.7	3.1	3.8	5.1	7.4	7	4.1	4.6	6.3	8.2
	vELS	c8	12.3	6.4	9.1	10.5	14.2	13.4	6.7	9.2	12	17.4	11.4	6.1	8.2	9.9	13	12.1	3.9	9.3	11.7	14.9
ER [%]-	ELS	c8	5	7.4	0.3	3.2	9.9	6.2	8	−0.5	6.3	12.3	3.9	6.6	0.3	3.2	6.5	−0.6	3.7	−3	−0.7	2.3
Difference	cELS	c8	2.3	6.2	−0.4	2.1	6.5	2.7	6.8	−0.4	2.5	6.7	1.9	5.8	−0.7	2.1	5.1	−0.7	4.6	−2.6	0	1.9
	vELS	c8	7.3	10.8	0.2	6.2	12	8.9	12.5	1.1	9.9	18	6	9.1	0	6.2	9.8	−0.5	5.4	−4	−0.4	2.4
TFS	ELS	c8	36.9	3.8	34.5	36.6	39.3	37.2	3.6	35.3	37.1	39.6	36.6	3.9	34	36.5	38.5	36.2	3.7	34.1	37.1	39
Ipsilateral	cELS	c8	16.3	1.7	15.5	16.4	17.4	16.5	1.6	15.3	16.6	17.6	16.3	1.8	15.6	16.4	17.3	15.8	1.8	14.9	15.9	16.8
[mm^2^]	vELS	c8	20.5	2.6	18.8	20.1	22.4	20.8	2.6	19.3	21.1	22.4	20.3	2.6	18.4	19.8	22.5	20.5	2.5	18.8	20.9	21.9
TFS	ELS	c8	37.3	3.9	35.3	37.1	39.4	37.1	3.5	35	36.6	39.4	37.5	4.2	35.3	37.1	39.4	36.6	3.3	34.2	37.1	37.9
Contralateral	cELS	c8	16.2	1.8	15.2	16.1	17.3	16.3	1.4	15.3	16.2	17.1	16.2	2.1	15.1	15.9	17.8	16.1	1.6	14.9	15.8	17.2
[mm^2^]	vELS	c8	21.1	2.7	19.5	20.8	22.7	20.8	2.5	18.5	20.7	22.3	21.3	2.8	19.6	20.8	22.8	20.4	2.2	18.7	20.4	22.3
**(D) 3D-quantification**																						
			**Mean**	**Std**	**25%**	**50%**	**75%**	**Mean**	**Std**	**25%**	**50%**	**75%**	**Mean**	**Std**	**25%**	**50%**	**75%**	**Mean**	**Std**	**25%**	**50%**	**75%**
Ipsilateral	ELS	c8	23.5	8.4	17.7	20.9	30.1	26	9	17.8	26.9	32.1	21.2	7.2	16.9	19.8	23.4	16.1	5.6	12.1	15.4	18.5
[mm^3^]	cELS	c8	6.8	3.5	4.4	5.4	9	7.8	3.6	4.8	7.2	10.7	5.9	3.1	4.4	4.9	6.9	5	2.4	3.5	4.6	6
	vELS	c8	16.6	6.1	12.4	15.3	20.5	18.2	7	13	19.3	22.2	15.3	4.9	12.2	14.8	17.2	11.1	3.6	9	11.6	12.9
Contralateral	ELS	c8	17.2	4.6	14.4	16.3	20	17.4	4.5	14.9	17.4	20	17.1	4.7	14.4	16.2	19.9	16.2	3.9	13.2	16.5	19.2
[mm^3^]	cELS	c8	5	2	3.7	4.6	6.4	5.3	2.3	3.7	4.8	6.5	4.8	1.8	3.6	4.6	6	5	2	3.2	4.7	6.1
	vELS	c8	12.2	3.8	9.8	11.4	14.3	12.2	3.2	10	12.8	14.2	12.3	4.4	9.6	11.4	14.4	11.2	3	8.8	11.7	13.1
AI [%]	ELS	c8	13.4	16.1	1.1	10	22.8	17.5	17.0	2.9	17.5	32.0	9.7	14.5	0.3	10	18.8	1.9	15.9	12.3	0.6	5.9
	cELS	c8	11.9	25.8	5.4	7.94	29.4	17.6	27.1	3.1	20.8	31.7	6.9	23.9	10.6	2.5	26.6	0.8	23.9	15.1	6.8	13.1
	vELS	c8	13.5	16.9	1.1	12.7	25.9	16.4	18.3	0.52	18.4	32.9	10.7	15.3	1.5	9.1	14.7	1.6	17.12	12.0	3.8	7.4
ER [%]	ELS	c8	8.5	2.8	6.4	7.5	10.8	9.3	2.9	7	9.6	11.9	7.7	2.5	6	7.3	8.9	5.9	1.6	4.8	5.9	6.5
Ipsilateral	cELS	c8	7	3.3	4.7	6	9.8	8	3.3	5.2	7.4	11	6.2	3	4.6	5.2	6.5	5.5	2.2	4	5.1	6.6
	vELS	c8	9.2	3.1	7	8.8	11.3	10	3.4	8.4	10.6	12.7	8.5	2.7	6.2	8.1	10.2	6.2	1.7	5.4	6.2	7
ER [%]	ELS	c8	6.2	1.3	5.4	6.1	6.9	6.2	1.3	5.6	6.4	7.2	6.1	1.3	5.3	6	6.7	6	1.2	4.8	6	6.8
Contralateral	cELS	c8	5.3	1.9	4	5.1	6.2	5.4	2	4	5	6.7	5.2	1.9	4	5.1	5.7	5.4	2.1	3.9	5	7
	vELS	c8	6.7	1.8	5.4	6.3	7.8	6.7	1.7	5.3	7.2	7.9	6.6	1.9	5.6	6.2	7.2	6.2	1.4	5.4	6.5	7
ER [%]-	ELS	c8	2.3	2.8	0.2	1.3	4	3.1	2.9	0.7	2.5	6.1	1.6	2.4	−0.1	1.3	2.7	0	1.7	−1	0	0.8
Difference	cELS	c8	1.8	3.5	−0.6	1	3.8	2.6	3.6	−0.5	2.4	5	1	3.3	−0.6	0.1	2.1	0.1	2.4	−1.4	0	1.6
	vELS	c8	2.5	3	0	1.9	4.7	3.3	3.3	0.3	3.2	6.3	1.9	2.6	−0.1	1.6	2.9	−0.1	1.9	−1.3	−0.5	0.9
TFS	ELS	c8	275.7	29.2	258	271.4	295	276.9	26.8	259.2	275	296.4	274.8	31.5	252.8	271.4	295	267.4	37.2	251	271.1	291
Ipsilateral	cELS	c8	95.2	12.2	86.7	93.4	103	96.7	12.3	87.5	96.7	105.4	93.9	12.2	85.8	93.4	102	88.6	14.4	80.5	88.2	98.4
[mm^3^]	vELS	c8	179.9	20.1	166	182	192	178.6	17.6	166.5	176.3	192.7	181	22.2	165.7	182	192	178.7	27.1	166	178.6	195
TFS	ELS	c8	276.5	31.2	257	269.1	298	277.3	30	256.8	275.1	300.9	275.8	32.5	258.6	269.1	298	269.9	25.7	248	270.6	290
Contralateral	cELS	c8	94.8	13.3	86.2	93	103	95.9	13.1	86.7	95	104.2	93.8	13.6	83	90.6	102	91.3	12.5	80.3	89.8	96.3
[mm^3^]	vELS	c8	182.4	20.6	171	183.3	193	181.3	19.5	171.5	178.2	192.7	183.4	21.7	171.4	183.4	193	178.6	17.8	168	180.7	190

### Influences of Signal Quality on ELS Quantification Methods (i)

- The signal intensity (SI) of each region of interest significantly (FWE-corrected, *p* < 0.05) depended on Gd dosage (range: 0.08–0.28 ml/kg; mean ± std: 0.17 ± 0.05 ml/kg; 48% of subjects got 1 dose, 12% 1.5 doses; 40% got 2 doses of 0.1 ml/kg) and Gd time delay (range: 2 h and 51 min to 5 h and 20 min; mean ± std: 4 h and 24 ± 25 min; 25% are between 3 and 4 h, 50% are between 4 and 4 h and 31 min and the remaining 25% were longer). However, the effect sizes (eta-squared) were small (5–12%).
- The mean SNR (range: 24.8–130.49; mean ± std: 64.82 ± 20.64) was significantly related to Gd dosage and time delay (FWE-corrected, *p* = 0.006), but only 6.8% of the total variance (*r*-squared) could be explained. If looked at separately, Gd dosage (4.2%, FWE-corrected, *p* = 0.03) and Gd time delay (5.3%, FWE-corrected, *p* = 0.02) explained even less of the variance. See Figure 2 for an overview of the minor influence of the iMRI acquisition parameters on SNR.
- The mean SNR was significantly different between the MD and HC group (*p* < 0.05). SNR asymmetry between left and right ear was not significantly related to Gd dosage, Gd time delay, or Gd dosage × Gd time delay.
- SQ gradings and 2D- or 3D- quantifications were not significantly related to Gd dosage, Gd time delay, Gd dosage × Gd time delay interaction, SI or SNR (FWE corrected, *p* ≤ 0.05). There were some simple significant relationships (*p* < 0.05 uncorrected) for the iMRI variables with Gd dosage and SNR, but all these relationships were small in effect size (around 0.5–5% omega squared).

### Interrelations Between ELS Quantification Methods (ii)

- Inter-rater SQ gradings (R1-3) were statistically homogeneous, as were 2D- and 3D- quantification values including ipsi- and contralateral or cochlea and vestibulum.
- Inter-rater SQ gradings (R1-3) were highly matched in the vestibular (v) and cochlear (c) part of the inner ear for all subjects (HC, MD) and slightly less for MD only. The results can be viewed in Table 4 (column SQ) and suggest a high but imperfect reproducibility due to remaining variability.
- Inter-threshold (c6, c8, and c10) 2D- and 3D-quantification was highly concordant. These results can be viewed in Table 4 (column 2D and 3D) and indicate an almost perfect agreement over VOLT thresholds with a basically perfect reproducibility.
- SQ grades correlated strongly with 2D-quantification values (range of correlation from 0.3 to 0.7) and 3D-quantification values (range of correlation from 0.3 to 0.7). The correlations of 2D- and 3D-quantification values with SQ grades was mainly driven by the MD group, due to the higher variability within the group, compared to HC group which did not vary much in grades or 2D- and 3D-quantification values (cp. Figure 3, plots on the left and in the middle).
- 2D- and 3D-quantification correlated substantially (range of correlation from 0.3 to 0.8) for the total inner ear, cochlea, and vestibulum on both the ipsilateral and contralateral side. However, there were no significant correlations of the ipsilateral with the contralateral sides (cp. Figure 3, plots on the right). *AI*_SQ_ (asymmetry-index of SQ quantification) correlated significantly (range of correlation from 0.3 to 0.7) with *AI*_2D_ and *AI*_3D_ (asymmetry-indices of 2D- and 3D-quantification) except for the cochlear *AI* in the 2D- and 3D-quantifications in the c6-cutoff ($cAI_{2D}^{c6}$ and $cAI_{3D}^{c6}$, cp. Figure 3).
- Inter-rater SQ grading differences did not differ strongly between R1-3. Figure 4 shows the results in more detail. For the vestibular part, the percentage of ratings that agreed, i.e., showed zero differences, was 54.6% (R2-R1), 50% (R3-R1), and 67.6% (R3-R2), while the percentage of differences of maximally one grade apart was 85.2% (R2-R1), 90.7% (R3-R1), and 98.2% (R3-R2). For the cochlear part, the percentage of ratings that agreed was 53.7% (R2-R1), 53.7% (R3-R1), and 71.3% (R3-R2), while the percentage of differences of maximally one grade apart was 88.9% (R2-R1), 90.7% (R3-R1), and 97.2% (R3-R2).
- Inter-threshold 2D- and 3D-quantification measures were statistically homogenous and showed group differences for each threshold.
- Clinical variables correlated with symmetry parameters derived from SQ grading and 2D- or 3D-quantification values such as the asymmetry index (*AI)* or the plain ELH difference between ipsilateral and contralateral side for the inner ear, vestibulum, and cochlea. The *AI* worked for un-normalized data and its results were comparable to the normalized data, while the pure differences between ipsilateral and contralateral sides were only useful when the data was first normalized to the fraction ER [%] of the total fluid space (TFS, cf. legend on standard values). This indicates that relative proportions of both ears, and the relative size of ELH are most useful for predicting quantitative clinical data from iMRI measures. Fittingly, vestibular AI for the 3D-quantification data explained 35% of the variance of the number of attacks in the 3 months prior to the examination and another 16% of variance could be explained by the AI for the 3D-quantification data of the whole of the inner ear (vestibular and cochlear parts combined). A more detailed clinical study and discussion can be found in another work (57).

**Table 4:** Interrelations between ELS quantification methods.

	**Vestibulum**			**Cochlea**		
	**3D**	**2D**	**SQ**	**3D**	**2D**	**SQ**
**(A) MD** **+** **HC**						
W	0.99	0.99	0.74	0.99	0.97	0.70
*p*-Value	6e-180	7e-175	5e-26	1e-180	3e-124	1e-20
*F*-Value	238.1	213.3	5.7	242.2	69.9	4.7
**(B) MD**						
W	0.99	0.99	0.71	0.99	0.98	0.62
*p*-Value	4e-125	7e-129	2e-15	2e-128	2e-103	8e-09
*F*-Value	238.5	268.5	4.9	264.5	120.3	3.2

*W = Kendall's coefficient of concordance between multiple rankings. Its value ranges from 0 to 1, unity being attained for perfect agreement between rankings*.

### Influences on Signal Quality (iii)

- There were significant differences in SI due to the presence of ELH in the following ROIs: cochlear basal turn [*p*_(t−*test*)_ = 0.0009 and *p*_(rank−sum)_ = 0.0003], apex cochleae [*p*_(t−*test*)_ = 0.002 and *p*_(rank−sum)_ = 0.001], hSCC [*p*_(t−*test*)_ = 0.038 and *p*_(rank−sum)_ = 0.022], and pSCC [*p*_(t−test)_ = 0.046 and *p*_(rank−sum)_ = 0.018]. Generally, higher ELH 3D-quantification values had higher SI values. However, due to a significant spread, SI could not distinguish the presence of ELH from the absence of ELH (tested by means of the split of SI values based on defining “absence of ELH” as an SQ grading equal to zero and “presence of ELH” as all grades higher than zero). Fittingly, the opposite approach (splitting ELH values by SI brightness) did not show significant differences. For an overview, please see Figure 5. The signal intensity SI was significantly different between the MD and HC group for both ROIs in the cochlear basal turn, but not for the cochlear apex, hSCC, or pSCC (*p* < 0.05 FWE). The group differences in iMRI variables between the MD and HC groups persisted after removing effects of Gd dosage, time delay, and SNR, indicating that iMRI assessment was not significantly affected by the differences in Gd dosage, time delay, and SNR in the present dataset.
- SNR was not influenced significantly by the presence or absence of an ELH. Selecting SNR values for all SQ grades = 0 (“absence of ELH”) and comparing them with the remaining SNR values (where SQ grades >0, “presence of ELH”) led to two-sample *t*-test *p* = 0.99 and two-sample rank-sum test *p* = 0.94. Furthermore, comparing the SNR values for low ELH values (3D-quantification values below the median) with SNR values for high ELH values (3D-quantification values above the median) did not show any significant differences in SNR (two-sample *t*-test *p* = 0.31 and two-sample rank-sum test *p* = 0.45). Analog to this, splitting ELH values due to low SNR values vs. high SNR values did not result in significant differences [*p*_(t−*test*)_ = 0.66 and *p*_(rank−sum)_ = 0.47 on the ipsilateral side and *p*_(t−testtest)_ = 0.2 and *p*_(rank−sum)_ = 0.16 for the contralateral side]. For an overview, see Figure 5.

### Standard Values

- Areas and volumes were normalized according to their TFS (total fluid space/surface) and can be viewed in Figure 6.
- Our calculations showed that the chosen threshold did not change the group differences between MD and HC. The grading-specific 2D- and 3D-quantification values, the TFS values and resulting ratios can be seen in Figures 6, 7. Furthermore, we show the relationship of 2D- and 3D-quantification for the vestibular and cochlear part broken down by SQ grades in Figure 8 and Table 5. While grades increase, one can observe that 2D- as well as 3D-quantification increased.
- ELH 3D-quantification values (see also Figure 6): The medians (ipsilateral, contralateral) of the vestibular data were (15 mm^3^, 11 mm^3^) for the MD group and (12 mm^3^, 12 mm^3^) for the vestibular healthy control (HC) group. The medians of the cochlear data were (5.4 mm^3^, 4.6 mm^3^) for the MD group and (4.6 mm^3^, 4.7 mm^3^) for the HC group.
- Total fluid space (TFS) 3D-quantification values of the vestibular and cochlear part of the inner ear [in mm^3^] that were used for normalizing the data of each individual to generate ELS ratio (ER) [%], the percentage of the TFS occupied by the ELH. The medians (ipsilateral, contralateral) of the vestibular TFS data were (182 mm^3^, 183 mm^3^) for the MD group and (179 mm^3^, 181 mm^3^) for the HC group. The medians of the cochlear TFS data were (93 mm^3^, 93 mm^3^) for the MD group and (88 mm^3^, 90 mm^3^) for the HC group.
- Therefore, the medians (ipsilateral, contralateral) of the vestibular ER [%] data were (8.8%, 6.3%) for the MD group and (6.3%, 6.5%) for the HC group. The medians of the cochlear ER [%] data were (6.0%, 5.1%) for the MD group and (5.1%, 5.0%) for the HC group.
- ELH 2D-quantification values (see also Figure 7): The medians (ipsilateral, contralateral) of the vestibular data were (3.6 mm^2^, 2.2 mm^2^) for the MD group and (2.3 mm^2^, 2.4 mm^2^) for the vestibular healthy control (HC) group. The medians of the cochlear data were (1.2 mm^2^, 0.8 mm^2^) for the MD group and (1.1 mm^2^, 1.0 mm^2^) for the HC group.
- Total fluid surface (TFS) 2D-quantification values of the vestibular and cochlear part of the inner ear, in [mm^2^] that were used for normalizing the data of each individual to generate ELS ratio (ER) [%], the percentage of the TFS occupied by the ELH. The medians (ipsilateral, contralateral) of the vestibular TFS data were (20.1 mm^2^, 20.8 mm^2^) for the MD group and (20.9 mm^2^, 20.4 mm^2^) for the HC group. The medians of the cochlear TFS data were (16.4 mm^2^, 16.1 mm^2^) for the MD group and (15.9 mm^2^, 15.8 mm^2^) for the HC group.
- Therefore, the medians (ipsilateral, contralateral) of the vestibular ER [%] data were (19.2%, 10.5%) for the MD group and (10.7%, 11.7%) for the HC group. The medians of the cochlear ER [%] data were (7.9%, 5.3%) for the MD group and (6.3%, 6.3%) for the HC group.

**Table 5:** SQ-specific 2D- and 3D-quantification of the ELS.

			**Grade 0**	**Grade 1**	**Grade 2**	**Grade 3**
			**No hydrops**	**Mild hydrops**	**Marked hydrops**	**Severe hydrops**
			**Mean ± std (min–max)**	**Mean ± std (min–max)**	**Mean ± std (min–max)**	**Mean ± std (min–max)**
**(A) 2D-quantification**						
c6	Inner ear [mm^2^]	R1-3	2.15 ± 1.32 (0.25–4.81)	3.57 ± 1.81 (0.19–6.81)	5.91 ± 1.92 (2.44–9.75)	6.31 ± 2.75 (3.19–8.38)
	Cochlea [mm^2^]	R1-3	0.56 ± 0.40 (0–1.31)	0.96 ± 0.77 (0–3)	1.29 ± 0.72 (0.06–2.56)	1.78 ± 0.13 (1.69–1.88)
	Vestibulum [mm^2^]	R1-3	1.88 ± 1.24 (0.13–4.25)	2.67 ± 1.62 (0–5.63)	4.18 ± 1.68 (1.13–8.63)	5.89 ± 2.04 (2.56–8)
c8	Inner ear [mm^2^]	R1-3	3.08 ± 1.54 (0.63–6.00)	4.77 ± 2.07 (0.5–8.5)	7.59 ± 2.20 (3.25–11.13)	8.19 ± 3.32 (4.38–10.44)
	Cochlea [mm^2^]	R1-3	0.89 ± 0.51 (0.25–1.94)	1.38 ± 0.92 (0.13–3.88)	1.76 ± 0.89 (0.19–3.31)	2.59 ± 0.04 (2.56–2.63)
	Vestibulum [mm^2^]	R1-3	2.45 ± 1.39 (0.31–5.19)	3.45 ± 1.84 (0.13–6.81)	5.34 ± 1.84 (1.88–9.44)	7.13 ± 2.30 (3.19–9)
c10	Inner ear [mm^2^]	R1-3	4.46 ± 1.87 (1.44–7.63)	6.01 ± 2.27 (1.19–10.63)	9.07 ± 2.28 (4.38–12.75)	9.77 ± 3.26 (6.06–12.19)
	Cochlea [mm^2^]	R1-3	1.31 ± 0.54 (0.56–2.13)	1.84 ± 1.00 (0.44–4.44)	2.25 ± 1.00 (0.44–3.88)	3.22 ± 0.04 (3.19–3.25)
	Vestibulum [mm^2^]	R1-3	3.21 ± 1.64 (0.56–6.38)	4.24 ± 1.98 (0.56–8)	6.25 ± 1.98 (2.19–10.13)	8.10 ± 2.56 (3.81–10.31)
TFS	Cochlea [mm^2^]	R1-3	15.54 ± 1.49 (12.63–18.63)	16.64 ± 1.20 (14.13–9.63)	16.59 ± 2.48 (9.63–21.88)	16.50 ± 1.24 (15.63–17.38)
	Vestibulum [mm^2^]	R1-3	19.45 ± 2.92 (15.00–24.63)	20.63 ± 2.66 (15.63–25.5)	20.78 ± 2.13 (17.31–25.19)	21.74 ± 3.03 (18–25.5)
c6	Inner ear ER [%]	R1-3	6.10 ± 3.74 (0.85–13.68)	9.54 ± 4.72 (0.64–17.76)	15.71 ± 4.90 (5.58–25.12)	16.99 ± 7.20 (8.73–21.93)
	Cochlea ER [%]	R1-3	3.51 ± 2.43 (0.00–7.78)	5.67 ± 4.33 (0–16.78)	7.93 ± 4.35 (0.29–14.96)	10.80 ± 0.01 (10.79–10.8)
	Vestibulum ER [%]	R1-3	9.50 ± 6.43 (0.82–21.79)	12.78 ± 7.61 (0–25.07)	20.09 ± 7.76 (5.96–40.83)	27.02 ± 8.95 (12.97–37.10)
c8	Inner ear ER [%]	R1-3	8.78 ± 4.36 (2.12–17.05)	12.75 ± 5.29 (1.71–21.55)	20.20 ± 5.64 (6.91–28.50)	22.10 ± 8.95 (11.99–29.00)
	Cochlea ER [%]	R1-3	5.65 ± 3.09 (1.44–11.48)	8.22 ± 5.14 (0.78–21.38)	10.83 ± 5.34 (0.86–18.90)	15.75 ± 0.91 (15.11–16.4)
	Vestibulum ER [%]	R1-3	12.49 ± 7.18 (2.08–26.60)	16.46 ± 8.52 (0.79–30.36)	25.71 ± 8.55 (9.93–44.67)	32.72 ± 10.1 (16.14–41.74)
c10	Inner ear ER [%]	R1-3	12.70 ± 5.20 (4.88–21.67)	16.10 ± 5.75 (4.06–27.96)	24.16 ± 5.91 (9.30–31.88)	26.35 ± 8.60 (16.61–32.90)
	Cochlea ER [%]	R1-3	8.34 ± 3.33 (3.94–15.25)	10.95 ± 5.58 (2.23–24.49)	13.84 ± 6.11 (2.57–21.99)	19.57 ± 1.74 (18.35–20.80)
	Vestibulum ER [%]	R1-3	16.34 ± 8.41 (3.75–32.69)	20.31 ± 9.01 (3.54–37.85)	30.07 ± 9.20 (11.59–47.93)	37.15 ± 10.9 (19.30–47.83)
**(B) 3D-quantification**						
c6	Inner ear [mm^3^]	R1-3	11.17 ± 3.85 (2.70–19.11)	14.52 ± 4.97 (7.48–27.97)	22.78 ± 5.54 (13.50–32.76)	23.13 ± 8.61 (13.50–30.10)
	Cochlea [mm^3^]	R1-3	3.15 ± 1.23 (0.76–6.20)	4.65 ± 2.93 (1.5–12.91)	6.49 ± 2.51 (2.89–11.80)	10.62 ± 2.62 (8.77–12.46)
	Vestibulum [mm^3^]	R1-3	8.27 ± 3.25 (1.38–14.53)	10.43 ± 3.63 (4.52–17.96)	14.81 ± 4.68 (5.56–25.53)	18.23 ± 5.43 (10.19–24.39)
c8	Inner ear [mm^3^]	R1-3	16.59 ± 5.24 (4.81–26.95)	20.73 ± 6.03 (11.02–36.47)	30.61 ± 6.78 (19.10–44.54)	31.16 ± 10.2 (19.78–39.56)
	Cochlea [mm^3^]	R1-3	4.58 ± 1.60 (1.31–8.11)	6.57 ± 3.51 (2.67–16.14)	8.73 ± 3.00 (4.59–14.70)	13.39 ± 3.22 (11.11–15.67)
	Vestibulum [mm^3^]	R1-3	12.29 ± 4.39 (2.56–20.86)	14.78 ± 4.48 (7–23.98)	20.12 ± 5.42 (9.48–30.26)	24.53 ± 6.49 (14.78–30.87)
c10	Inner ear [mm^3^]	R1-3	23.06 ± 6.63 (7.97–35.78)	27.87 ± 6.83 (15.34–44.95)	38.85 ± 8.05 (24.97–56.85)	39.68 ± 11.5 (27.13–49.73)
	Cochlea [mm^3^]	R1-3	6.38 ± 2.03 (2.05–10.05)	8.74 ± 3.96 (4.02–18.84)	11.12 ± 3.38 (6.19–18.26)	16.15 ± 4.24 (13.16–19.15)
	Vestibulum [mm^3^]	R1-3	17.10 ± 5.45 (4.64–27.53)	19.78 ± 5.19 (9.52–29.81)	25.78 ± 6.15 (14.06–36.55)	31.28 ± 7.41 (20.41–38.59)
TFS	Cochlea [mm^3^]	R1-3	90.0 ± 10.6 (68.96–105.82)	95.4 ± 11.2 (74.25–117.52)	99.90 ± 14.1 (75.5–124.47)	97.1 ± 17.6 (84.63–109.56)
	Vestibulum [mm^3^]	R1-3	178.6 ± 18.3 (149.7–219.7)	181.2 ± 23.3 (132.2.0.0.221.7)	176.8 ± 18.2 (140.3–227.3)	190.4 ± 11.2 (175.0–199.6)
c6	Inner ear ER [%]	R1-3	4.12 ± 1.23 (1.15–6.25)	5.32 ± 1.83 (2.90–9.82)	8.05 ± 1.68 (4.46–10.73)	8.25 ± 2.84 (4.98–9.94)
	Cochlea ER [%]	R1-3	3.49 ± 1.48 (1.10–8.19)	4.76 ± 2.67 (1.49–12.02)	6.55 ± 2.46 (2.59–11.11)	10.87 ± 0.72 (10.36–11.38)
	Vestibulum ER [%]	R1-3	4.57 ± 1.63 (0.87–7.07)	5.82 ± 2.04 (2.43–9.62)	8.30 ± 2.26 (3.57–13.20)	9.51 ± 2.51 (5.60–12.22)
c8	Inner ear ER [%]	R1-3	6.13 ± 1.66 (2.04–8.82)	7.60 ± 2.21 (4.44–12.81)	10.83 ± 2.04 (6.18–14.11)	11.12 ± 3.32 (7.29–13.15)
	Cochlea ER [%]	R1-3	5.07 ± 1.86 (1.90–10.71)	6.76 ± 3.17 (2.78–15.03)	8.81 ± 2.90 (3.88–13.85)	13.71 ± 0.83 (13.13–14.3)
	Vestibulum ER [%]	R1-3	6.79 ± 2.17 (1.62–10.14)	8.24 ± 2.54 (3.99–12.84)	11.30 ± 2.53 (6.09–16.61)	12.81 ± 2.96 (8.12–15.47)
c10	Inner ear ER [%]	R1-3	8.53 ± 2.07 (3.38–11.71)	10.20 ± 2.47 (6.19–15.79)	13.75 ± 2.42 (8.28–17.95)	14.16 ± 3.61 (10.0–16.25)
	Cochlea ER [%]	R1-3	7.04 ± 2.25 (2.98–13.26)	9.03 ± 3.52 (4.21–17.54)	11.24 ± 3.29 (5.36–16.24)	16.51 ± 1.37 (15.55–17.48)
	Vestibulum ER [%]	R1-3	9.48 ± 2.66 (2.94–13.39)	11.01 ± 2.95 (5.99–15.97)	14.50 ± 2.79 (9.03–20.09)	16.35 ± 3.29 (11.22–19.42)

## Discussion

This methodological study with 108 participants (75 MD, 33 HC) focused on comparability and parametrization of different ELS quantification methods (SQ grading of three raters, 2D- or 3D-quantification of three cutoffs) used in iMRI and their (i) interrelations with subtle variations in data acquisition protocols (that influence SNR or SI); (ii) correlations to each other, clinical symptoms, or neurophysiological testing; and (iii) the influence of ELH on signal quality. The results were as follows: (i) Within the range of 0.1–0.2 mmol/kg (mean ± std: 0.16 ± 0.05 mmol/kg) Gd dosage and a 3 h 41 min to 5 h 19 min (mean ± std: 4 h 39 min ± 25 min) time delay, SQ gradings, and 2D- or 3D-quantifications were independent of signal intensity (SI) and signal-to-noise ratio (SNR), but they were found to be significantly related to Gd dosage and time delay themselves. (ii) The ELS quantification methods used were highly reproducible across raters (SQ gradings) or thresholds (2D- and 3D-quantification), although 3D-quantifications showed least variability in comparison to 2D-quantifications and SQ gradings. The relative proportions of both ears, and the relative size of ELH proved to be most useful for predicting quantitative clinical data from iMRI measures. (iii) ELH size significantly influenced SI but not SNR. In contrast, SI could not predict ELH size. In the following, results (i–iii) will be discussed.

### Within a Specific Dosage and Time Delay Range ELS Quantification Methods Remain Independent of Signal Intensity (i)

The 3D fluid-attenuated inversion recovery (3D- FLAIR) imaging used has high sensitivity to low concentrations of Gd-based contrast agents (GBCA) in fluid compared with conventional T_1_-weighted imaging (58). In particular, the heavily T_2_-weighted 3D-FLAIR imaging with a long effective echo time is very sensitive to subtle T1 shortening and can detect low concentrations of GBCAs in the perilymphatic space after intravenous administration of a single dose of GBCA (18, 59, 60). In the tested Gd dosage and Gd time delay range (see above), at most weak influences on SNR and no influence on ELS quantification methods were found. It can therefore be assumed that, although Gd dosage and Gd time delay certainly have an influence on iMRI quality parameters, the sweet spot for ELH quantification by iMRI is within the range of the tested parameters. These results tie in well with earlier studies that showed strongest enhancement in 3D FLAIR sequences between 3 and 6 h (61), and optimally 4 h (62) after intravenous administration of 0.1 mmol/kg gadolinium diethylenetriaminepentaacetic acid (standard dose Gd-DTPA, Magnevist®), or 4.5 h (63) after intravenous administration of a standard dose gadoteridol (0.1 mmol/kg Gd-HP-DO3A, ProHance®), double dose (0.2 mmol/kg) Gd-HP-DO3A (64) or triple dose (0.3 mmol/kg) of Gd-DTPA-BMA, Omniscan® (65).

Another feature of the good performance within the chosen ranges may be the homogeneous distribution of the contrast agent in the entire volume of the inner ear (66, 67).

Further improvement of SNR and visualization in terms of rapid, morphological enhancement for analysis of the temporal and spatial distribution in the PLS of the inner ear can be achieved through careful selection of MR sequences (59, 68), combination (69, 70), and post-processing (14) of MR sequences, MR Gd complex (71), MR coil, and MR field strength (72).

### Is There a Hierarchy Within ELS Quantification Methods? (ii)

In line with the only comparative methodological study of ELS quantification methods published to date in 11 participants (9 patients and 2 healthy controls) (26), SQ grading and 2D- or 3D- quantification methods were found to be reliable and useful for the diagnosis of endolymphatic hydrops. However, the degree of reliability based on comparisons between raters or thresholds increased from SQ grading to 2D- and again to 3D-quantification methods. The increase in repeatability corresponds to the decrease in dependency of human decision (visually > specific slice in 2D > whole volume in 3D) and increase of automatization and data points (semi-quantitative < area < volume).

Another aspect that makes relying solely on SQ grading tricky is the comparability of methods between different research groups, besides inter-rater disparities. SQ grading conventions (cf. Table 1) vary in grading resolution from three [in cochlea (19–21, 23) and vestibulum (19, 24, 25)] to four steps [in cochlea (22, 24, 25) and vestibulum (21–23)]. Accordingly, not all ELH grade results in cochlea or vestibulum correspond to each other due to the usage of different conventions [as an example grade 1 in (19, 20, 24)], or not at all [as an example (73)]. Based on either manually drawn (28, 74) regions of interest (ROIs) or a convolutional neural network (CNN) segmentation (32), 2D quantification methods already offer an increased comparability and variability of information. However, the comparability of the results remains limited by the slice selection for the calculation of the ratio and the differing slices emerging from slice planning or MRI setup (sequence type, slice thickness, slice resolution). Concerning these issues, 3D-quantification can be a solution (no slice selection, independent of slice planning) or at least an improvement (sequence type, slice thickness, slice resolution). In addition, more information (data points) enables better fitting of diagnostic and clinical parameters (75). Yet here, too, methodological variations affect reproducibility and availability of results. The critical points are the segmentation of the inner ear from the background [manually (29), via atlas (76, 77), or CNN (31); (Ahmadi et al., under review)] and the ELS and PLS from the TFS [manually (26), semi-automatic (29), automatic (31)], as well as the availability of the software solutions [commercial (26, 28, 29, 78) vs. open source (31)]. The less human-dependent and the more automated, the more reproducible the method in most cases. Therefore, the usefulness of the available quantification methods depends on its intended application. While visual SQ grading is highly useful in a clinical setting, automated 3D-quantification seems most suitable for research.

### ELS Patterns in MD, HC (ii)

Significant differences between groups could especially be found for the ipsilateral (or affected) side of the MD group vs. HC group, as was already shown for 4-point [cochlea: (24, 25)] and 3-point [cochlea: (79); vestibulum: (80); sacculum (81)] ELS SQ grading, 2D-quantification [cochlea: (82); vestibulum: (82)] and 3D-quantification [cochlea: (30, 57); vestibulum: (30, 57)] results.

Clinical variables correlated highest with symmetry parameters derived from SQ grading and 2D- or 3D-quantification values such as the asymmetry index (*AI*) or the plain ELH difference between ipsilateral and contralateral side for the inner ear, vestibulum, and cochlea. Recent studies using ELS asymmetry indexes confirm this inclination (57). A more detailed clinical study and discussion can be found in another work (76). To date, correlations were found for SQ grades 3-point [electrocochleography (EcochG) (83, 84)] and 4-point ordinal cochlear scale [PTA (24–26, 79); auditory symptoms (85, 86); disease duration (24, 79); but not for the glycerol test (25)]. Furthermore, correlations were found for 3-point ordinal vestibular scale [cVEMP-side difference (SD) (24); PTA (11, 25, 87); oVEMP-amplitudes (88), but not with VOG during caloric stimulation SD (24, 89) or the glycerol test (25)]. SQ correlations coincided with 2D-quantification [cochlea: PTA (82); vestibulum: SP/AP ratio of ECoG (82)] and 3D-quantification [cochlea: PTA (26); vestibulum: duration of illness >30 months (26), side difference in response to caloric irrigation (57)] correlation results.

### ELH Extent Influences Signal Intensity in the Basal Cochlear Turn (iii)

Zhang et al. (90) investigated 19 MD patients following double-dose iMRI and found that the signal intensity ratio of the cochlear basal turns in the affected ear was significantly higher than in the unaffected ear and that there was a positive correlation between the signal intensity ratio of the cochlear basal turn and the grades of cochlear and vestibular hydrops in the affected ear. The SNR was assessed and calculated manually according to (91) using the signal in perilymph of both cochlear basal turns and noise in coplanar circular 50 mm^2^ ROIs in the cerebellum. The interpretation of these findings was that increased permeability of the blood-labyrinth barrier (higher SNR) may play a role in the process of endolymphatic hydrops in MD.

The results of the current study suggest, however, a general pathophysiological effect tied to the extent of the ELH and not MD as a pathology, since higher ELH 3D-quantification values had higher signal intensity (SI) values in the cochlear basal turn, apex cochlea, and hSCC ROIs. Within MD, SI only in the cochlear basal turn was significantly higher on the ipsilateral side when compared to the contralateral side. The SI was generally different between the MD and HC groups, indicating an effect of ELH also on signal presentation. SNR differed between the MD and HC groups; however, the effect was small and the group differences in ELH were not significantly affected by SNR, indicating that the group differences are a persistent effect of the underlying condition and not related to the imaging settings that were used in the current study.

### Normalization and Standardization of ELS Values

Clinical variables correlated better and more correctly with relative (AI) or normalized values [to the fraction ER [%] of the total fluid space]. This indicates that relative proportions of both ears, and the relative size of ELH are most useful for predicting quantitative clinical data from iMRI measures (57).

However, to date not many iMRI ELS values have been published in absolute (26, 30, 92) and relative sizes (26, 28, 85, 93–95); those that have been published were mostly group-specific but not grade-specific, and one grade-specific but relative (26). In Table 5, 2D- and 3D-quantifications, relative, and normalized to TFS are presented.

### Recommendations for Future iMRI Studies

The following methodological recommendations for future studies can be derived from the present work and the current available literature:

- MR setup: Improved hybrid of reversed image of positive endolymph signal and native image of positive perilymph **s**ignal (iHYDROPS-Mi2) (15) or 3D-real inversion recovery (3D-real IR) (28, 96), highest possible MRI field strength (72), smallest possible isotropic voxel size, deep learning reconstruction denoising (14) if applicable.
- MR measurement: 4 h ±30 min after single-dose (0.1 mmol/kg) intravenous application (64) of Dotarem (Gd-DOTA, 100% morphological enhancement) or Gadovist (Gd-Do3A, 88% morphological enhancement) (71).
- SQ grading: 4-point ordinal scale (0: no hydrops, 1: mild hydrops, 2: marked hydrops, 3: severe hydrops) for cochlear and vestibular SQ grading (cp. Table 1), preferably with a level of evaluation reconstructed to distinctive anatomical fixpoints.
- Scalar ELS values: 3D-quantification, optimally using algorithm-based segmentation of both the TFS (Seyed-Ahmad et al., under review); (76, 77) and ELS (31, 32) should be included. 2D-quantification if 3D-quantification is not available. Reported values should be normalized for TFS size.
- Correlations with clinical variables should include both ears and are most promising in symmetry parameters, such as asymmetry-indices for un-normalized data and relative size ELS for normalized data.

### Methodological Limitations and Outlook

There are methodological limitations in the current study that need to be considered in the interpretation of the data. First, despite the comparatively wide range of contrast agent dosage and delay time within this study, the results should be (to some degree) considered specific to the study's MR settings (MR sequence, MR contrast agent, intravenous application). Second, despite the extensive analyses within this study, it was not possible to try all, but only representations of the methods used in this study [SQ following Figure 1 and (22), 2D- and 3D-quantification using VOLT (31)]. Third, the study lacks histological confirmation of endolymphatic hydrops. However, the *in-vivo* acquisition of histological specimens in Menière's disease is currently not possible. Fourth, the size of the control group (*n* = 33) was small in comparison to the MD group (*n* = 105). However, due to findings of signal intensity in the dendate nucleus and globus pallidus on unenhanced T1-weighted MR images (97–99) that are still under investigation, measurements were restricted to inpatients of the Department of Neurology that underwent MRI with a contrast agent as part of their diagnostic workup and agreed to undergo iMRI sequences after 4 h.

## Conclusion

The current comparative methodological study has shown that: (1) A Gd dosage of 0.1–0.2 mmol/kg after 4 h ± 30 min Gd time delay will provide sufficient SNR when using recommended MR sequences and contrast agents. (2) An agreed upon clinical SQ grading classification including a standardized level of evaluation reconstructed to anatomical fixpoints is needed to provide unambiguous comparability between labs. (3) 3D-quantification methods of the ELS using algorithm-based segmentation of the TFS and ELS seem to be best suited for research purposes. Correlations with clinical variables should include both ears and ELS values reported relative or normalized to size. (4) The presence of ELH increases signal intensity in the basal cochlear turn weakly, but cannot predict the presence of ELH.

## Data Availability Statement

The original contributions presented in the study are included in the article/supplementary material, further inquiries can be directed to the corresponding author/s.

## Ethics Statement

The studies involving human participants were reviewed and approved by Ethics Committee of the Medical Faculty of the Ludwig-Maximilians-Universität, Munich, Germany. The patients/participants provided their written informed consent to participate in this study.

## Author Contributions

RB: conception and design of the study, analysis of the data, and drafting the manuscript. JG, EK, SB-B, and BE-W: acquisition, analysis of the data, and drafting of the manuscript. MD: conception and design of the study, drafting the manuscript, and providing funding. VK: conception and design of the study, acquisition, analysis of the data, drafting the manuscript, and providing funding. All authors contributed to the article and approved the submitted version.

## Conflict of Interest

The authors declare that the research was conducted in the absence of any commercial or financial relationships that could be construed as a potential conflict of interest.

## Abbreviations

±: standard deviation
2D: two-dimensional
3D: three-dimensional
AR: area ratio
AI: asymmetry index
B: bilateral
c: cochlea
contra: contralateral
CEMD: central eye movement disorder
CISS: constructive interference in steady-state
d: definite
Diff-ER: ELS ratio of the side difference
DL: deep learning
ELH: endolymphatic hydrops
ELS: endolymphatic space
ER: ELS ratio
FLAIR: fluid-attenuated inversion recovery
Gd: gadolinium
GBCA: Gd-based contrast agents
Gd-DOTA: gadoteric acid (trade name: Dotarem®)
Gd-Do3A: gadobutrol (trade name: Gadovist®)
Gd-DTPA-BM: gadodiamide (trade name: Omniscan®)
Gd-DTPA: gadopentetic acid (trade name: Magnevist®)
Gd-HP-DO3A: gadoteridol (trade name: ProHance®)
GLM: general linear model
GRAPPA: generalized auto-calibrating partially parallel acquisition
HC: healthy controls
HIT: head-impulse test
iMRI: delayed intravenous gadolinium-enhanced MRI of the inner ear
ipsi: ipsilateral
iv: intravenous
L: left
R: right
MD: Meniere's disease
minEn: minimum energy statistic
MMD: maximum mean discrepancy
MRI: magnetic resonance imaging
n: number
p: possible
ROI: region-of-interest
std: standard deviation
SNR: signal-to-noise ratio
SVV: subjective visual vertical
TFS: total fluid space
v: vestibulum
vHIT: video-oculography during head-impulse test
VM: vestibular migraine
VOG: video-oculography.
